# Applications and Performance of Machine Learning Algorithms in Emergency Medical Services: A Scoping Review

**DOI:** 10.1017/S1049023X24000414

**Published:** 2024-10

**Authors:** Ahmad Alrawashdeh, Saeed Alqahtani, Zaid I. Alkhatib, Khalid Kheirallah, Nebras Y. Melhem, Mahmoud Alwidyan, Arwa M. Al-Dekah, Talal Alshammari, Ziad Nehme

**Affiliations:** 1. Department of Allied Medical Sciences, Jordan University of Science and Technology, Irbid, Jordan; 2. Department of Emergency Medical Services, Prince Sultan Military College for Health Sciences, Dhahran, Saudi Arabia; 3. Department of Public Health and Family Medicine, Faculty of Medicine, Jordan University of Science and Technology, Irbid, Jordan; 4. Department of Anatomy, Physiology and Biochemistry, Faculty of Medicine, The Hashemite University, Zarqa, Jordan; 5. Kernel research and data analytics center, Irbid, Jordan; 6. Department of Emergency Medical Care, College of Applied Medical Sciences, Imam Abdulrahman Bin Faisal University, Dammam, Saudi Arabia; 7. Ambulance Victoria, Doncaster, Victoria, Australia; 8. School of Public Health and Preventive Medicine, Monash University, Melbourne, Victoria, Australia

**Keywords:** ambulance, artificial intelligence, deep learning, Emergency Medical Services, machine learning

## Abstract

**Objective:**

The aim of this study was to summarize the literature on the applications of machine learning (ML) and their performance in Emergency Medical Services (EMS).

**Methods:**

Four relevant electronic databases were searched (from inception through January 2024) for all original studies that employed EMS-guided ML algorithms to enhance the clinical and operational performance of EMS. Two reviewers screened the retrieved studies and extracted relevant data from the included studies. The characteristics of included studies, employed ML algorithms, and their performance were quantitively described across primary domains and subdomains.

**Results:**

This review included a total of 164 studies published from 2005 through 2024. Of those, 125 were clinical domain focused and 39 were operational. The characteristics of ML algorithms such as sample size, number and type of input features, and performance varied between and within domains and subdomains of applications. Clinical applications of ML algorithms involved triage or diagnosis classification (n = 62), treatment prediction (n = 12), or clinical outcome prediction (n = 50), mainly for out-of-hospital cardiac arrest/OHCA (n = 62), cardiovascular diseases/CVDs (n = 19), and trauma (n = 24). The performance of these ML algorithms varied, with a median area under the receiver operating characteristic curve (AUC) of 85.6%, accuracy of 88.1%, sensitivity of 86.05%, and specificity of 86.5%. Within the operational studies, the operational task of most ML algorithms was ambulance allocation (n = 21), followed by ambulance detection (n = 5), ambulance deployment (n = 5), route optimization (n = 5), and quality assurance (n = 3). The performance of all operational ML algorithms varied and had a median AUC of 96.1%, accuracy of 90.0%, sensitivity of 94.4%, and specificity of 87.7%. Generally, neural network and ensemble algorithms, to some degree, out-performed other ML algorithms.

**Conclusion:**

Triaging and managing different prehospital medical conditions and augmenting ambulance performance can be improved by ML algorithms. Future reports should focus on a specific clinical condition or operational task to improve the precision of the performance metrics of ML models.

## Introduction

Machine learning (ML) is a valuable and increasingly common tool for modern health care systems. Across many medical areas, ML algorithms have aided health care professionals to provide an accurate diagnosis for a disease, make an appropriate clinical decision for a case, and predict an outcome for a treatment intervention.^1,2^ However, many of the applications of ML algorithms have focused on improving the safety and quality of care for patients within the in-hospital setting.^2–4^ The potential benefits of ML applications in the prehospital setting are not yet well-established. In-depth knowledge regarding ML applications on prehospital patient care is important for identifying effective technologies and providing guidance for ML-driven research in this specific domain.

Personnel from Emergency Medical Services (EMS) often respond to prehospital cases requiring a rapid response time, early recognition of a life-threatening condition, and timely treatments. Across these time-sensitive tasks, there is evidence that ML algorithms have the potential to augment EMS performance. For example, the response time of a dynamic ambulance re-deployment strategy has been reduced by 1.5 minutes using ML algorithms in reference to conventional methods that are manually designed.^5^ In addition, dispatchers of EMS who used a clinical decision support algorithm to recognize out-of-hospital cardiac arrest (OHCA) were able to capture more cases within a shorter time interval than those who used the conventional “No-No-Go” approach.^6^ Despite this, according to the 2015-2020 report for approving ML-based medical products in the United States and Europe, none of the EMS-guided ML applications have been translated into a real product.^7^

To date, few reviews have summarized the application of ML in emergency medicine, but none is exclusive to prehospital emergency medical care or ambulance service.^8–10^ While many EMS-guided ML applications have been developed, it is difficult to determine the value of each application in the absence of consolidated evidence. This review, therefore, aimed to systematically review and describe the literature on EMS-guided ML applications in both clinical and operational purposes, and further to describe the characteristics of ML algorithms and their performance.

## Methods

The study approach involved conducting a scoping review of ML applications in EMS, complemented by a quantitative synthesis of their performance metrics. This review was conducted in accordance with the Preferred Reporting Items for Systematic Reviews and Meta-Analyses for Scoping Reviews (PRISMA-ScR) guidelines.^11^ The protocol of this review is registered with PROSPERO (ID: CRD42021271256).

### Search Strategy

The literature was systematically reviewed from inception until January 9, 2024 across four databases, including: Medline (US National Library of Medicine, National Institutes of Health; Bethesda, Maryland USA); Scopus (Elsevier; Amsterdam, Netherlands); CINAHL (EBSCO Information Services; Ipswich, Massachusetts USA); and Computers and Applied Sciences (EBSCO Information Services; Ipswich, Massachusetts USA). A comprehensive search strategy was created using relevant keywords, phrases, text terms, and subject headings (Supplementary Material S1 Table; available online only). The search strategy was designed to capture all reports related to EMS, ambulance, or paramedic and ML algorithms, deep learning, or neural networks. Search results were imported to the EndNote reference software (Version X9; Clarivate Analytics; Philadelphia, Pennsylvania USA). After removing duplicates, search results were then imported to Covidence software (Covidence; Melbourne, VIC, Australia) for managing and streamlining the review.^12^

### Study Selection

Two independent reviewers (AA and ZA) initially screened the titles and abstracts for eligibility. At this stage, a study was deemed pertinent if it was written in English; original research; peer-reviewed trials, observational, or experimental studies; and utilized a data-driven methodology involving at least one ML algorithm aimed to classify or predict an EMS-specific outcome related to either clinical or operational applications specific to EMS use.

For a study to be classified under clinical application, the implemented ML algorithm must have been engineered with the aim of augmenting the predictive or classifying capabilities concerning a clinical condition, a clinical intervention, or a potential clinical outcome. For operational applications, the ML algorithm must have been geared towards enhancing the operational efficiency of an EMS system’s resources such as optimizing, streamlining, or improving the use of EMS resources.

Conference proceedings, news, case reports, editorial letters, commentaries, reviews, and non-English reports were excluded. Studies that have used EMS data to aid emergency department and in-hospital outcomes were also excluded. After screening the titles and abstracts, a full-text screening was carried out by the two reviewers (AA and ZA) for final inclusion. Any disagreement was resolved through consensus.

### Data Collection

A standardized extraction form was developed to collect relevant data from the included studies. Baseline characteristics such as the first author, year of publication, study objective and design, location, duration, data source, and the number of cases were extracted. Information related to ML algorithms such as the number and types of ML algorithms used, input features, output outcomes, and model performance were also extracted. Additionally, the values of the performance metrics were extracted. This includes the values of the area under the receiver operating characteristic curve (AUC), accuracy, sensitivity, specificity, and positive predictive value. One reviewer extracted the relevant data (ZA), and this was cross-checked by a second reviewer (AA). Disagreements were resolved through consensus.

### Data Synthesis

The applications of ML algorithms across EMS studies were dichotomized into clinical and operational domains. Study characteristics such as the year of publication, geographic region, study design, data source, and number of ML algorithms employed across these domains were summarized using descriptive statistics. Numerical variables were, as appropriate, reported as medians and ranges and categorical variables were reported as counts and percentages.

The studies were then classified within the clinical domain into subdomains based on the medical condition, including OHCA, cardiovascular diseases (CVDs), trauma, and others (ie, including unspecified medical conditions). Similarly, the studies were classified within the operational domain into subdomains, and this was based on the operational task, including ambulance allocation (ie, ambulance demand forecasting and relocation), deployment (ie, ambulance dispatching), detection (ie, ambulance recognition by image or siren capturing), route optimization (ie, route selection), and quality assurance (ie, quality indicators or documentation assessment). All subdomains were assessed, using descriptive statistics as described above, against the following variables: number of studies, clinical tasks, number of cases, the number and type of input features, methods of ML algorithm, and performance metrics.

The values of performance metrics were evaluated by estimating the unweighted medians and ranges. The performance metrics estimated included AUC, accuracy, sensitivity, and specificity for the best ML algorithm as declared by individual studies. STATA statistical software, version 16.0 (Stata Corp; College Station, Texas USA) was used to carry out all statistical analyses.

## Results

**Figure 1.**
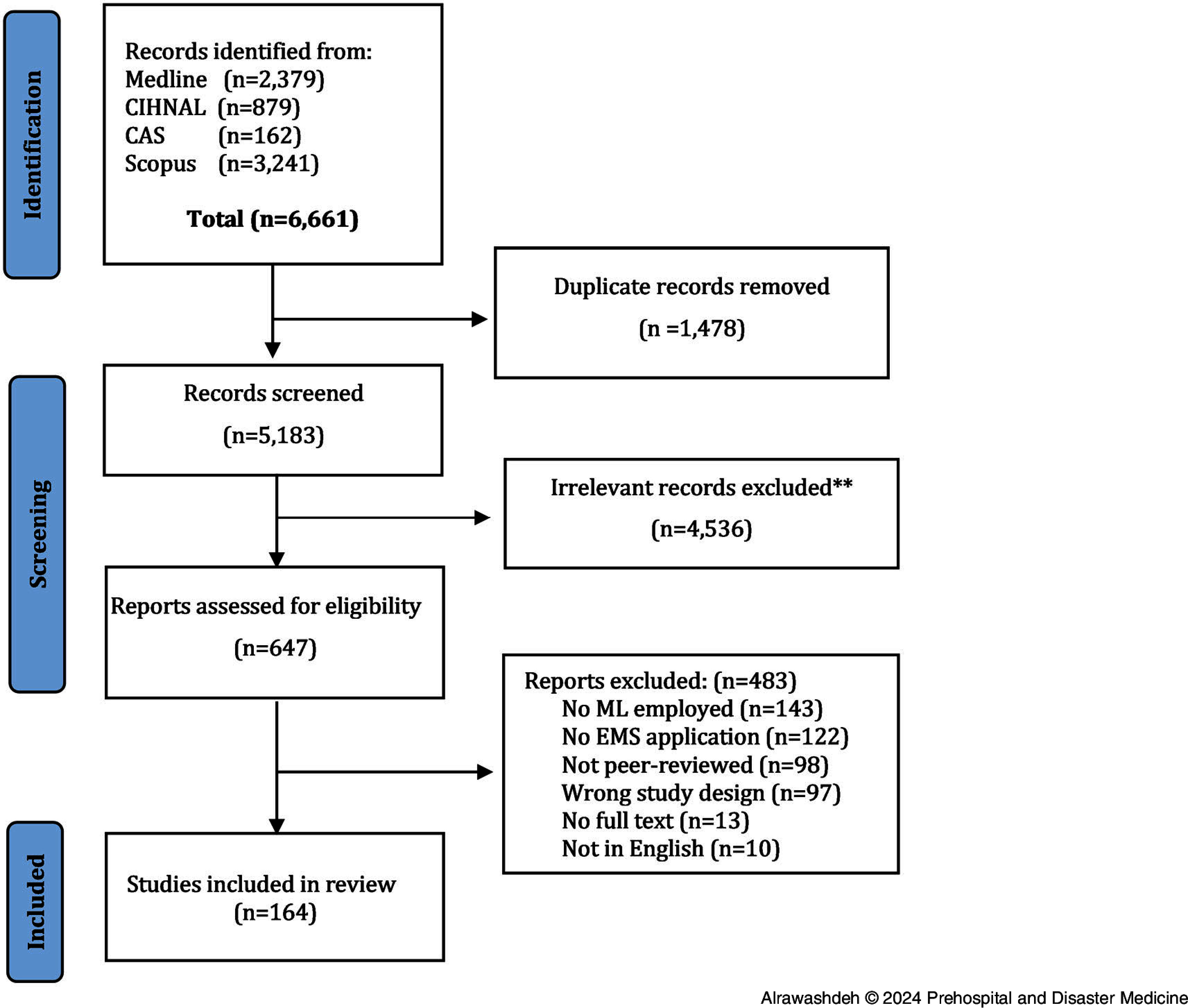
PRISMA Flow Chart. Abbreviations: ML, machine learning; EMS, Emergency Medical Services; CAS, Computer & Applied Sciences.

The search strategy identified 6,661 records, of which 1,478 were duplicates and 5,183 were excluded in the title and abstract screening. A total of 647 full-text articles were retrieved and assessed for eligibility. Finally, a total of 164 studies were included in this review (Figure 1).

### Characteristics of Included Studies

**Figure 2.**
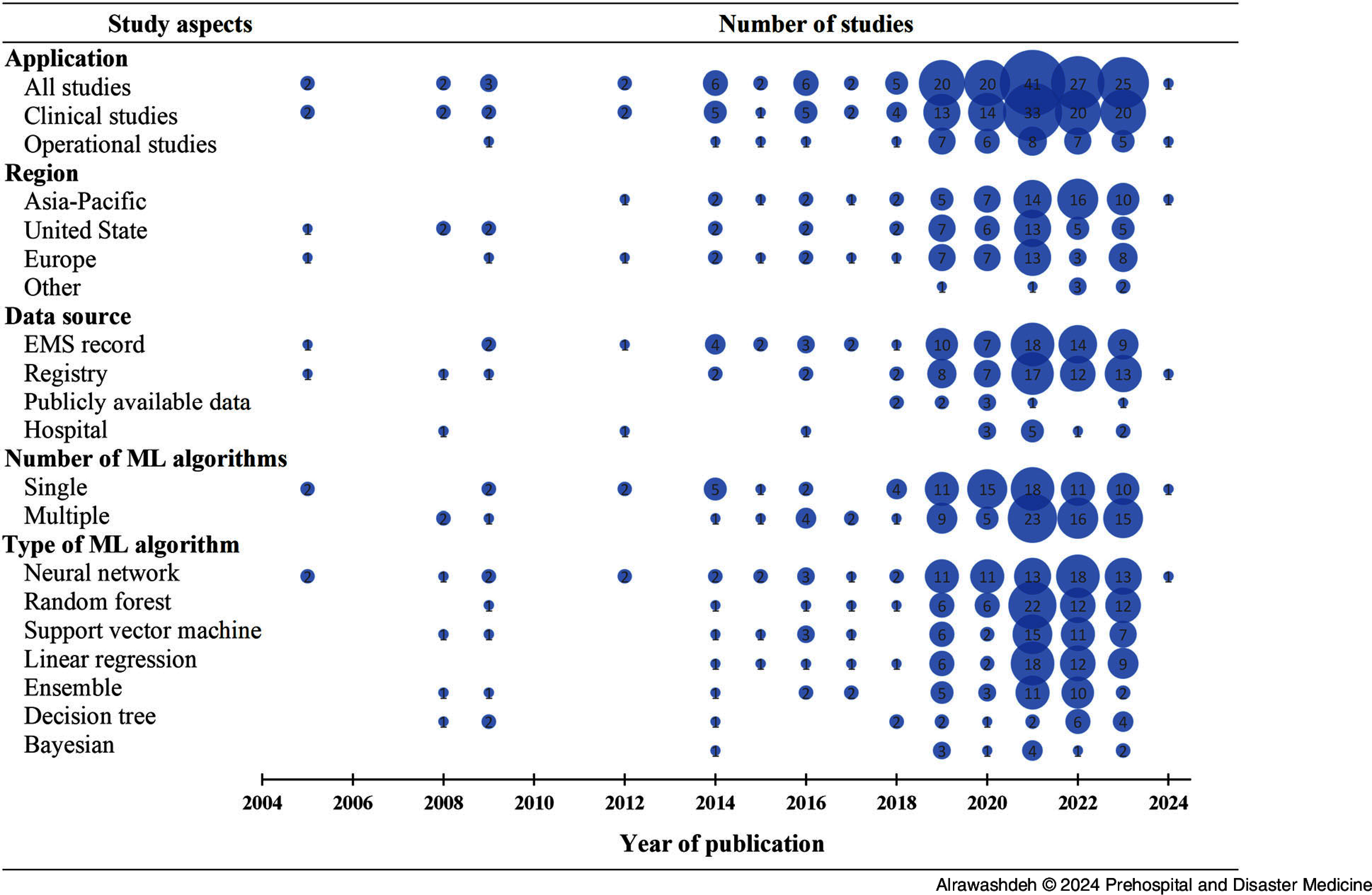
Distribution of the Included Studies Over the Years. Abbreviations: ML, machine learning; EMS, Emergency Medical Services.

Table 1 summarizes the characteristics of the included studies by domain. Overall (n = 164), the median year of publication was 2020 ranging from 2005 through 2024; the number of publications exponentially increased after 2017. Figure 2 shows the distribution of the included studies over the years for different characteristics of the included studies. More EMS-guided ML applications were reported in the Asia-Pacific region (37.2%) than those that were reported in Europe (29.3%) and the United States (28.7%); most studies were retrospective in design (94.5%) and the data were mainly derived from the EMS records (45.1%) and registries (40.9%); and 51.2% of the included studies used one ML algorithm, whereas 48.8% of them used more than one. More than two-thirds of the included studies deployed ML for a clinical application (125 studies; 76.2%), while 39 (23.8%) studies applied ML for an operational application. All included studies are listed and described in Table S2 and Table S3 in the Supplementary Material (available online only). Operational studies were reported mostly in the Asia-Pacific (51.3%), whereas the United States and Europe had a similar number of studies, with a predominant focus on clinical research.

**Table 1.** Baseline Characteristics of the Included Studies across the Two Domains

Study Characteristics	Overall	Domain	
		Clinical	Operational
**Number of Studies,** n (%)	164 (100)	125 (76.2)	39 (23.8)
**Year of Publication**, range	2005-2024	2005-2023	2009-2024
**Study Design,** n (%)			
Retrospective Observational	155 (94.5)	117 (93.6)	38 (97.4)
Prospective Observational	9 (5.5)	8 (6.4)	1 (2.6)
**Study Duration in Years,** ^a^ median (range)	3.0 (0.1-16)	3.0 (0.1-16)	3.0 (0.3-10)
**Region,** n (%)			
Asia-Pacific	62 (37.8)	42 (33.6)	20 (51.3)
United States	47 (28.7)	41 (32.8)	6 (15.4)
Europe	48 (29.3)	41 (32.8)	7 (17.9)
Others	7 (4.3)	1 (0.8)	6 (15.4)
**Data Source,** n (%)			
EMS Record	74 (45.1)	53 (42.4)	21 (53.9)
Registry	67 (40.9)	53 (42.4)	14 (35.8)
Publicly Available Data	9 (8.5)	5 (4.0)	4 (10.3)
Hospital	14 (8.5)	14 (11.2)	0 (0.0)
**Number of Used ML Algorithms,** n (%)			
Single	84 (51.2)	59 (47.2)	25 (64.1)
Multiple	80 (48.8)	66 (52.8)	14 (35.9)

Abbreviations: EMS, Emergency Medical Services; ML, machine learning.

a Reported in 125 studies (n = 104 for clinical domain and n = 21 for operational).

### Characteristics of ML Algorithms in the Clinical Domain

Table 2 presents the characteristics of EMS-guided ML algorithms applied in the clinical domain studies, categorized by medical conditions, including OHCA (n = 62 studies; 49.6%), CVDs (n = 19 studies; 15.2%), trauma (n = 24 studies; 19.2%), and others (n = 20 studies; 16.0%). The purpose of most studies in this domain was to primarily triage or diagnose a medical condition (50.0%), followed by predicting a clinical outcome (40.3%) and a clinical intervention (9.7%).

**Table 2.** Characteristics of EMS-Guided ML Applications across Studies in Clinical Conditions

	Total	OHCA	CVDs	Trauma	Others
**Number of Studies, n (%)**	125	62	19	24	20
**Purpose of the ML Algorithm, n (%)**					
Triage or Diagnose Conditions	62 (50.0)	22 (35.5)	18 (94.7)	11 (45.8)	11 (57.9)
Predict Clinical Interventions	12 (9.7)	2 (3.2)	1 (5.3)	4 (16.7)	5 (26.3)
Predict Clinical Outcomes	50 (40.3)	38 (61.3)	0 (0.0)	9 (37.5)	3 (15.8)
**Model Task**					
Classification	62 (49.6)	26 (41.9)	13 (68.4)	12 (50.0)	11 (55.0)
Prediction	63 (50.4)	36 (58.1)	6 (31.6)	12 (50.0)	9 (45.0)
**Number of Cases/Instances,** ^**a**^ median (range)	3,405 (37-8,981,181)	1,376 (100-177,883)	560 (48-3,178)	21,874 (37-8,981,181)	54,359 (1043-684,481)
**Number of Input Features,** ^**b**^ median (range)	19 (1-886)	16 (1-886)	23 (5-557)	15.5 (4-84)	17 (2-86)
**Types of Input Features**					
Clinical Findings	66 (53.7)	24 (40.0)	12 (63.2)	18 (75.0)	12 (60.0)
ECG Strips	42 (35.0)	36 (59.0)	5 (27.8)	1 (4.2)	0 (0.0)
Text Scripts	13 (11.0)	1 (1.7)	3 (16.7)	3 (12.5)	6 (35.3)
Audio Recordings	6 (5.1)	4 (6.8)	0 (0.0)	0 (0.0)	2 (11.8)
Image	1 (0.9)	0 (0.0)	0 (0.0)	0 (0.0)	1 (5.9)
Other	13 (11.0)	6 (10.2)	1 (5.9)	4 (16.7)	2 (11.1)
**ML Algorithm, n (%)**					
Neural Network	60 (48.0)	30 (48.4)	6 (31.6)	16 (66.7)	8 (40.0)
Random Forest	57 (45.6)	25 (40.3)	11 (57.9)	12 (50.0)	9 (45.0)
Logistic Regression	47 (37.6)	20 (32.3)	7 (36.8)	13 (54.2)	7 (35.0)
Support Vector Machine	43 (34.4)	23 (37.1)	7 (36.8)	9 (37.5)	4 (20.0)
Ensemble	33 (26.4)	16 (25.8)	5 (26.3)	6 (25.0)	6 (30.0)
Decision Tree	17 (13.6)	11 (17.7)	1 (5.3)	4 (16.7)	1 (5.0)
Bayesian	7 (5.6)	2 (3.2)	0 (0.0)	3 (12.5)	2 (10.0)
K-Nearest Neighbors	6 (4.3)	2 (3.2)	2 (10.5)	2 (8.3)	0 (0.0)
**Performance Metrics, n (%)**					
AUC	76 (60.8)	33 (53.2)	13 (68.4)	19 (79.2)	11 (55.0)
Accuracy	42 (33.6)	16 (25.8)	11 (57.9)	7 (29.2)	8 (40.0)
Sensitivity	73 (58.4)	38 (61.3)	15 (78.9)	12 (50.0)	8 (40.0)
Specificity	67 (53.6)	33 (53.2)	15 (78.9)	12 (50.0)	7 (35.0)
Not Mentioned, Not Available	7 (5.6)	4 (6.5)	1 (5.3)	0 (0.0)	2 (10.0)

Abbreviations: OHCA, out-of-hospital cardiac arrest; CVD, cardiovascular diseases; ECG, electrocardiography; AUC, area under the receiver operating characteristic curve; ML, machine learning.

a Number of cases/instances is missing in 23 studies.

b Number of input features is missing in 54 studies.

Among OHCA studies, the most frequent purposes were predicting a clinical outcome (n = 38; 61.3%) and detecting or triaging OHCA (n = 22; 35.5%). Almost all CVD studies employed ML for triage and diagnosis (n = 18; 94.7%). The purpose of most studies concentrating on trauma was to triage traumatic cases (n = 11; 45.8%) or to predict a clinical outcome (n = 9; 37.5%).

The median number of cases/instances for most studies within the clinical domain was 3,405 (n = 102 studies; range: 37-8,981,181). The sample size was larger among studies focusing on trauma and other medical conditions than studies focusing on OHCA and CVDs. When reported, the median number of input features for studies within the clinical domain was 19 (n = 71 studies; range: 1-886). The number of input features was larger for CVD studies compared to other clinical conditions. Among all clinical domain studies, the most frequent type of input features was clinical (ie, demographics, vitals, and findings of physical examination) obtained from EMS medical records (53.7%), followed by electrocardiograph (ECG) strips (35.0%), text scripts (11.0%), and audio recordings (5.1%). The majority of the studies focused on OHCA involved ECG input features (n = 36; 59.0%), while most studies related to all other conditions involved clinical features.

The most frequently employed ML algorithms were neural network (n = 60 studies; 50.0%), followed by random forest (n = 57 studies; 48.3%), logistic regression (n = 47 studies; 39.8%), and support vector machine (n = 43 studies; 36.4%). This distribution was similar within all clinical conditions. To evaluate the performance of the ML algorithms, the most frequently used performance metric was AUC (n = 76; 60.8%), followed by sensitivity (n = 73; 58.4%) and specificity (n = 67; 53.6%).

### Performance of ML Algorithms in the Clinical Domain

In the clinical domain, the median AUC for the best ML algorithms was 85.6% (n = 76 studies; range: 64.0%-99.9%). The median accuracy was 88.1% (n = 40 studies; range: 57.8%-99.2%), sensitivity was 86.0% (n = 73 studies; range: 38.8%-99.7%), and specificity was 86.5% (n = 67 studies; range: 11.0%-99.9%).

**Figure 3.**
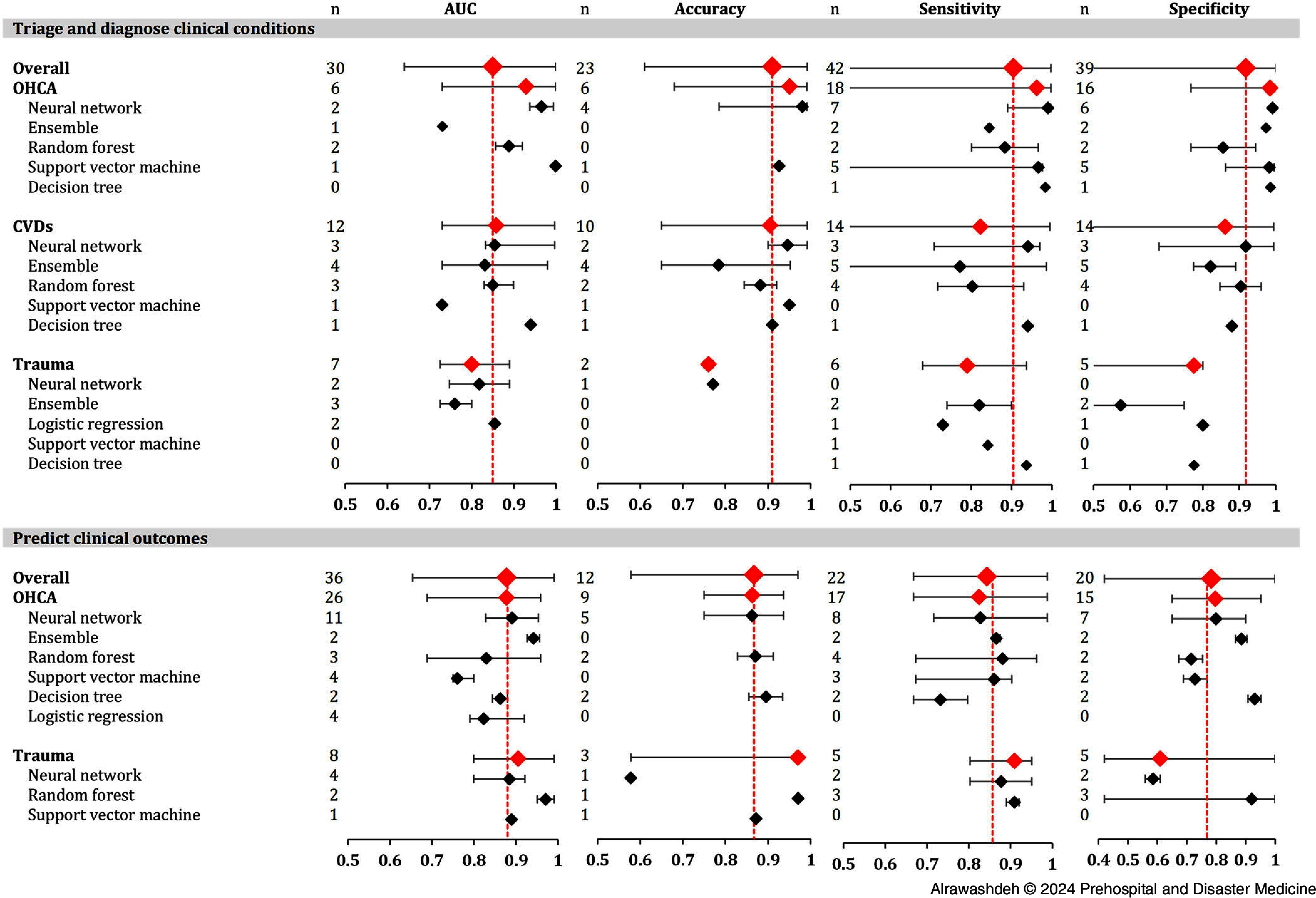
Median and Range of the Best Machine Learning (ML) Algorithm amid to Triage or Diagnose the Clinical Condition and Predict their Clinical Outcomes. Abbreviations: OHCA, out-of-hospital cardiac arrest; CVD, cardiovascular diseases; AUC, area under the receiver operating characteristic curve.

Figure 3 depicts the performance of the best ML algorithms employed to triage and diagnose clinical conditions and predict clinical outcomes across the clinical conditions and ML algorithms. Overall, the medians of accuracy, sensitivity, and specificity of the ML algorithms designed to triage and diagnose a clinical condition (≥90.0%) were higher than the algorithms designed to predict a clinical outcome (<90.0%).

The median values of most performance metrics for ML algorithms deployed to triage or diagnose clinical conditions were highest in the context of OHCA and lowest in traumatic emergencies. However, compared to algorithms predicting a clinical outcome after OHCA, algorithms predicting a clinical outcome after traumatic emergencies had a higher median AUC (87.3% versus 90.5%, respectively) and sensitivity (81.7% versus 91.0%), but lower accuracy (86.3% versus 87.6%) and specificity (80.3% versus 61.0%). Neural network, as well as ensemble algorithms, appeared to outperform other ML algorithms across clinical purposes and conditions.

### Characteristics of ML Algorithms in the Operational Domain

Table 3 presents the characteristics of EMS-guided ML algorithms applied in the operational domain studies, categorized by the operational task, including ambulance allocation (n = 21 studies), ambulance detection (n = 5 studies), ambulance deployment (n = 5 studies), route optimization (n = 5 studies), and quality assurance (n = 3 studies).

**Table 3.** Characteristics of EMS-Guided ML Applications across Studies in Operational Subdomains

	Total	Ambulance Allocation	Ambulance Deployment	Ambulance Detection	Route Optimization	Quality Assurance
**Number of Studies, n**	39	21	5	5	5	3
**Number of Cases,** ^a^median (Range)	11,899 (77-7,364,275)	9,766 (108-7,364,275)	78,777 (3,573-421,894)	4,230.5 (1,234-26,675)	48,681.5 (42,363-55,000)	7,054.5 (77-14,032)
**Number of Input Features,** ^b^median (Range)	10 (3-9,224)	7 (4-92)	18 (8-9,224)	–	9 (9-9)	6 (3-19)
**Source of Input Features, n (%)**						
EMS-Related	17 (43.6)	14 (66.7)	2 (40.0)	–	1 (20.0)	–
Spatiotemporal	13 (33.3)	9 (42.9)	1 (20.0)	–	3 (60.0)	–
Audio Recordings	3 (7.7)	–	–	3 (60.0)	–	–
Clinical Findings	10 (25.6)	4 (19.1)	2 (40.0)	–	1 (20.0)	3 (100)
Image	3 (7.7)	–	–	3 (60.0)	–	–
Traffic Reports	3 (7.7)	1 (4.8)	–	–	2 (40.0)	–
Other	6 (15.4)	5 (23.8)	1 (20.0)	–	–	–
**ML Algorithm, n (%)**						
Neural Network	24 (61.5)	13 (61.9)	4 (80.0)	5 (100)	2 (40.0)	–
Support Vector Machine	6 (15.4)	4 (19.1)	1 (20.0)	–	–	1 (33.3)
Ensemble	7 (17.9)	4 (19.1)	2 (40.0)	–	–	1 (33.3)
Random Forest	6 (15.4)	4 (19.1)	1 (20.0)	–	1 (20.0)	–
Decision Tree	4 (10.3)	2 (9.5)	1 (20.0)	–	1 (20.0)	–
K-Nearest Neighbors	4 (10.3)	2 (9.5)	–	–	1 (20.0)	1 (33.3)
Bayesian	5 (12.8)	3 (14.3)	1 (20.0)	–	–	1 (33.3)
Logistic Regression	5 (12.8)	3 (14.3)	–	–	–	2 (66.7)
**Performance Metrics, n (%)**						
AUC	6 (15.4)	2 (9.5)	–	1 (20.0)	1 (20.0)	2 (66.7)
Accuracy	17 (43.6)	5 (23.8)	3 (60.0)	4 (80.0)	4 (80.0)	1 (33.3)
Sensitivity	10 (25.6)	3 (14.3)	3 (60.0)	1 (20.0)	1 (20.0)	2 (66.7)
Specificity	6 (15.4)	3 (14.3)	1 (20.0)	1 (20.0)	–	1 (33.3)
Not Reported	18 (46.2)	15 (71.4)	1 (20.0)	1 (20.0)	1 (20.0)	–

Abbreviations: EMS, Emergency Medical Service; ML, machine learning; AUC, area under the receiver operating characteristic curve.

a Number of cases is missing in 19 studies.

b Number of input features is missing in 29 studies.

The overall median sample size was 11,899 (n = 20 studies; range: 77-7,364,275) and the largest sample sizes were reported in studies focusing on ambulance allocation and deployment. The overall median number of input features was 10 (n = 10 studies; range: 3-9,224) and the largest median was found in ambulance deployment studies (18.0; range: 8-9,224). Among studies within the operational domain, the most frequent types of input features were EMS-related data (n = 17 studies; 43.6%), spatiotemporal (n = 13 studies; 33.3%), clinical findings (n = 10 studies; 25.6%), audio recordings (n = 3 studies; 7.7%), and image (n = 3 studies; 7.7%).

The neural network was the most frequently employed ML algorithm (n = 24 studies; 61.5%) across all operational purposes. This was followed by ensemble algorithms (n = 7 studies; 17.9%), support vector machine (n = 6 studies; 15.4%), random forest (n = 6 studies; 15.4%), and decision tree (n = 4 studies; 10.3%). To evaluate the performance of the ML algorithms, accuracy (n = 17 studies; 43.6%) was the most frequently used performance metric, followed by sensitivity (n = 10 studies; 25.6%) and specificity (n = 6 studies; 15.4%). Notably, a total of 18 (46.2%) studies did not report the performance of their ML models.

### Performance of ML Algorithms in the Operational Domain

For the operational domain, the median AUC of the best ML algorithms was 96.1% (n = 6 studies; range: 89.8%-99.0%), accuracy was 90.0% (n = 17; range: 24.5%-98.7%), sensitivity was 94.4% (n = 10; range: 83.0%-99.5%), and specificity was 87.7% (n = 6; range: 66.8%-99.4%).

**Figure 4.**
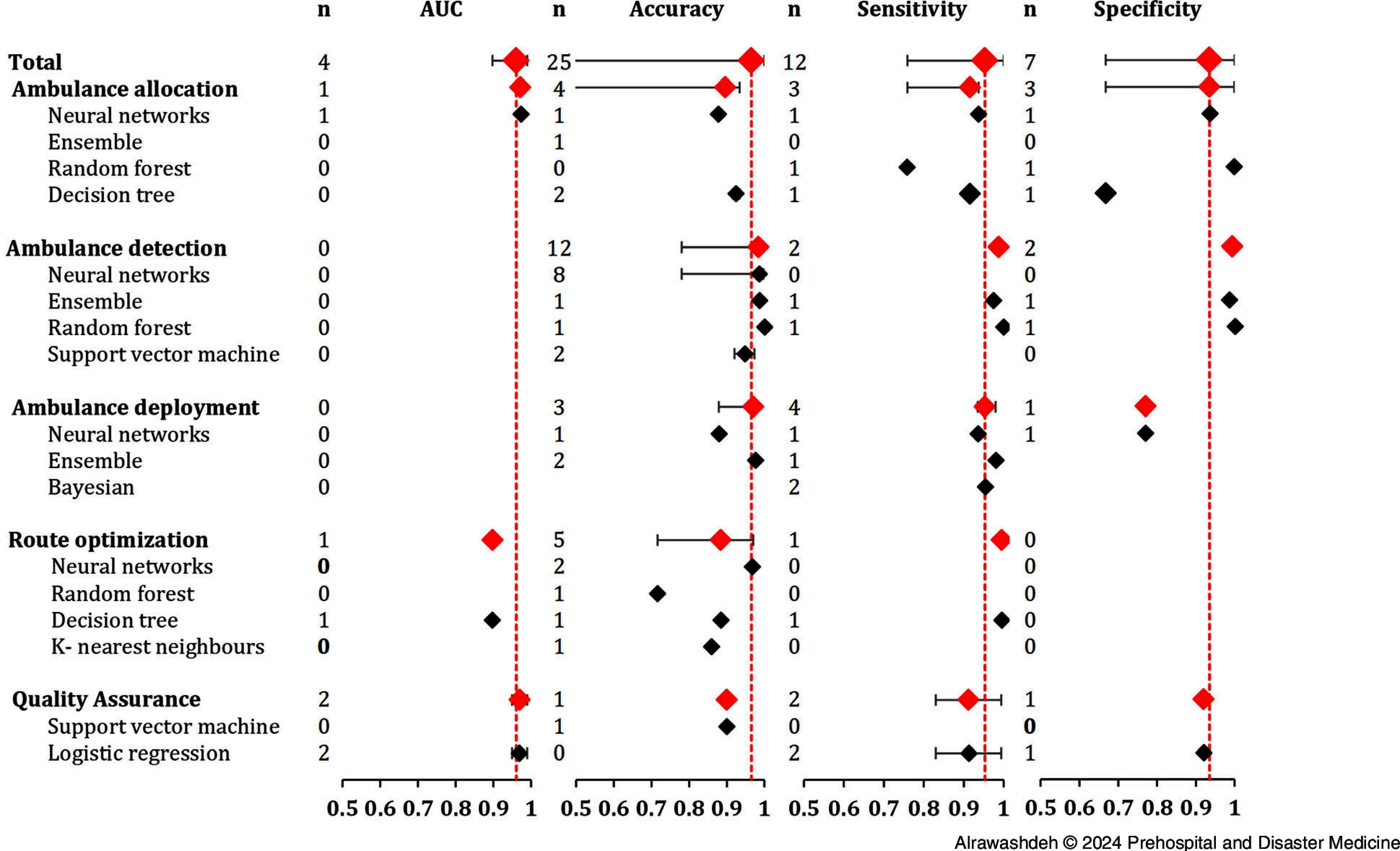
Median and Range of the Best Machine Learning (ML) Algorithm Categorized by Operational Tasks and Algorithm Type. Abbreviation: AUC, area under the receiver operating characteristic curve.

Figure 4 illustrates the performance of the best ML models categorized by operational tasks and types of ML algorithms. The medians of accuracy metrics across studies related to ambulance detection and deployment were higher than those related to ambulance allocation, route optimization, and quality assurance. The frequently applied algorithm was a neural network which, along with the ensemble, out-performed other algorithms to a certain level.

## Discussion

The journey of prehospital emergency care for a patient, including call receipt, dispatch, travel, triage, diagnosis, treatment, and transport to appropriate definitive care, is efficiently managed by multiple operational and medical components of EMS. This review showed that ML algorithms were implemented in almost all EMS components, including dispatch and communication system, first responders, ambulances and paramedics, medical oversight, and quality improvement. In addition, ML has the potential to augment the decision-making capabilities of EMS personnel in both clinical and operational domains or even supplement their judgment in specific aspects of EMS components. However, the integration of ML in EMS is still evolving, and further research, development, and validation may be needed to fully leverage its capabilities in clinical and operational applications. Considerable heterogeneity was observed in the included studies in terms of population characteristics, the ML algorithms employed, input features, and performance metrics which limited a more comprehensive review. The implications and performance of ML should be in-depth evaluated for each clinical condition, outcome, or operational task.

Several studies showed the potential of ML algorithms in improving emergency medical response to OHCA. More than 15 studies concentrated on implementing ML for classifying cardiac arrest ECG rhythms or detecting the return of spontaneous circulation aiming to improve resuscitation performance and provide timely guidance for delivering electrical defibrillation. Multiple ML algorithms exhibit outstanding performance (AUC > 0.90), demonstrating remarkable accuracy and practical applicability in the real-world context. For example, Jaureguibeitia, et al proposed novel deep learning architectures (AUC = 98.6%) for shock decisions using ECG segments as brief as one second, which likely shorten interruptions in resuscitation for rhythm analysis.^13^ Elola, et al also implemented deep neural networks for pulse detection using only the ECG (AUC = 86.2%),^14^ while Alonso, et al (AUC = 92.6%) combined ECG and thoracic impedance signals for superior pulse detection.^15^ Further, deep neural networks have demonstrated the ability to detect OHCA at the time of calling for help, arguably with greater accuracy and speed than the EMS telecommunicators.^6,16,17^

Predictive ML models were also developed for three clinical outcomes after OHCA, including the return of spontaneous circulation, survival, and neurological recovery. Among many ML models, deep neural networks achieved the highest performance (AUC = 0.84 to 0.90) in predicting the success of defibrillation using ECG features.^18–20^ Acknowledging the limited number of cases in most of these studies (n <500 cases), larger and more diverse samples of cases are likely essential to limit the risk of bias and to enhance the performance, reliability, and applicability of ML algorithms in predicting defibrillation success.^21^

Deep neural networks and ensemble ML algorithms performed exceptionally (AUC = 0.90 to 0.96) among others to predict survival and neurological recovery using prehospital clinical and EMS data.^22–24^ However, these predictive models exhibit considerable heterogeneity in quality, type of population, input and output features, and algorithm selection, potentially affecting generalizability. Additionally, the complexity and lack of transparency in certain ML algorithms might obstruct their integration into clinical practice.^21^ Future ML studies should discuss features attribution or explain the ML models output by using several methods such as Shapley Additive Explanation, Local Interpretable Model-agnostic Explanations, and integrated gradients.

Most ML models have also been shown to improve the detection of CVDs in the prehospital setting. A deep learning model achieved an impressive AUC score of 0.997 in detecting ST-elevation myocardial infarction (STEMI).^25^ This model has been deployed in a prehospital 12-lead ECG device and resulted in a shorter median time between first medical contact and hospital arrival (18.5 minutes), compared to the weighted mean estimated by a previous meta-analysis (41 minutes).^26^ Further, Bouzid, et al have developed a fusion model to detect non-STEMI using prehospital 12-lead ECG which significantly outperformed the performance of expert clinicians and commercial software.^27^ In addition, prehospital stroke diagnostic ML algorithms have been shown to be accurate in identifying and differentiating stroke using prehospital clinical presentation variables.^28–31^ Two studies have shown that neural network algorithms out-performed other available prehospital stroke prediction scales, such as the Prehospital Acute Stroke Severity scale and the Cincinnati Prehospital Stroke Severity scale.^28,29^ However, a larger number of parameters (ie, 18 variables) were included in these neural network models, which demand more effort and time to be collected by ambulance clinicians.

For traumatic conditions, ML models have been increasingly applied to primarily improve the triage decision by dispatchers, first responders, and ambulance clinicians in various out-of-hospital contexts (ie, general trauma, road accidents, and military). These models were developed either to classify traumatic patients by their level of severity (ie, Injury Severity Score)^32^ or to predict the level of treatment needed (ie, advanced life-saving interventions, trauma team activation, and critical care)^33,34^ or the clinical outcomes (ie, survival).^35^ The input features in most models were relatively limited and mainly consisted of demographics, vital signs, other clinical manifestations, or accident-related variables. Neural networks and ensemble techniques (random forest, XGBoosting) were frequently used and had the best performance compared to other models. Two studies found that binary logistic regression performed better than ML algorithms when classifying the severity of injuries.^32,36^ This is likely attributed to the limited number of input features in these studies. Overall, the contribution of ML seems to be promising in this context, but further validation is required to better understand the applicability of ML in supporting triage decisions and optimizing resource allocation.

Several ML models have been implemented to solve operational problems and augment the decision making of EMS personnel in operational tasks. The over-arching aims of the operational studies were to reduce ambulance travel time and deploy the appropriate level of prehospital care to the right patients. A variety of supervised, unsupervised, and reinforcement models have been used to improve EMS demand forecasting in different settings to optimize ambulance allocation strategies, and eventually reduce response times. The characteristics of ML models forecasting EMS demand varied in terms of time horizons (hourly, daily, weekly, and monthly demand) and input features (geospatial, temporal, metrological, and EMS data). Several studies aimed to facilitate ambulance passage through traffic congestion by either audio-based and/or visual-based detection. The audio-based models using random forest, support vector machine, and neural networks achieved AUC scores between 0.97-1.00.^37–40^ Visual-based models mainly used deep learning with variable performance (AUC = 0.78-1.00)^40–42^ while hybrid models incorporated both audio and image inputs and typically relied on deep learning yield strong results (0.86-0.98).^40,43^ The performance of these models indicates the viability of employing ML to detect ambulances and expedite their passage through intersections and traffic congestion.

Finally, the implementation of the developed ML models in real time was rarely discussed in the included studies. Several challenges such as the generalizability and explainability of these ML models may complicate their implication in clinical and real time. Several studies reported perfect discrimination, especially for operational purposes, which warrant a critical examination of the underlying data and methodologies employed. While perfect discrimination can be attributed to factors like over-fitting, reliance on internal validation, data leakage, or imbalanced datasets, it is imperative to consider the broader context of its applicability, generalizability, and potential limitations in real-world EMS scenarios. For example, the generalization of ML algorithms for ambulance detection using audio or image data may pose significant challenges due to the wide variation in ambulance sirens and morphologies across different EMS systems.

It is also important to acknowledge the complications and limitations associated with the retrospective nature of most included studies. Rich prehospital datasets are still necessary; however, they are likely challenging to obtain, owing to limitations in data quality and availability, patient privacy, and the heterogeneity of EMS system data frameworks.^44^ This necessitates collaborative efforts among researchers, EMS systems, and other health care institutions, as well as policymakers to establish data-sharing frameworks, best practices, and governance models that facilitate the responsible and impactful application of these technologies in the challenging and dynamic landscape of EMS. For better explainability, future ML studies should also discuss features attribution or explain the output of ML models by using available methods such as Shapley additive explanation, local interpretable model-agnostic explanations, and integrated gradients.^45^

## Limitations

This review has several limitations. Of note, this review may have some selection bias. It only included English peer-reviewed articles identified by the search strategy. The variation in the terminology of ML and EMS in the literature may also contribute to selection bias. The wide scope of this review may hinder proper summarization of the applications and performance of the ML in specific clinical conditions and operational tasks. For example, the approaches for features selection, model validation, and optimization used in the included studies were not detailed. Future systematic reviews could be done to quantify the use of ML in specific clinical conditions such as detecting OHCA or predicting the clinical outcomes after OHCA.

Also not examined was the risk of bias and quality of the included studies. This is attributed to the large number and the wide variation in the objectives and characteristics of the included studies. Although the performance of the ML algorithms performed well in most applications, the issue of risk of bias should be considered in most ML models, particularly within the analysis domain.^46,47^ Several studies had not reported all relevant details such as sampling, input features selection, validation procedures, and model performance, particularly for studies within the operational domain. These missing data also limit the overall analysis and the generalizability of the findings. Finally, this review did not assess whether the ML models deployed in the included studies had obtained regulatory approval from agencies such as the United States Food and Drug Administration (FDA; Silver Spring, Maryland USA) or other relevant authorities. This limitation should be acknowledged when considering the broader applicability of these models in clinical practice.

## Conclusion

This systematic review highlights the diverse applications and potential benefits of ML in EMS. Most studies focused on the use of ML algorithms to enhance the clinical domain, particularly in detecting and predicting the severity, treatment, and clinical outcome of patients with OHCA, CVDs, and trauma. The performance of ML models varied widely, indicating the need for further research and standardization of ML algorithms in EMS. Additionally, the review found that ML can improve the operational management of ambulance services, including optimizing ambulance allocations, reducing travel time, and enhancing resource utilization. This review is expected to provide useful benchmarks and implications for possible future research. Further research is needed to investigate the implementation and quality of ML in specific clinical conditions or operational tasks within the EMS.

## Supporting information

Alrawashdeh et al. supplementary material Alrawashdeh et al. supplementary material

## Supplementary Materials

To view supplementary material for this article, please visit https://doi.org/10.1017/S1049023X24000414
