# Applications and Performance of Machine Learning Algorithms in Emergency Medical Services: A Scoping Review – ADDENDUM

**DOI:** 10.1017/S1049023X24000724

**Published:** 2024-10

**Authors:** Ahmad Alrawashdeh, Saeed Alqahtani, Zaid I. Alkhatib, Khalid Kheirallah, Nebras Y. Melhem, Mahmoud Alwidyan, Arwa M. Al-Dekah, Talal Alshammari, Ziad Nehme

The following has been added to the Limitations section (beginning Page 9) of the above article:

Finally, this review did not assess whether the ML models deployed in the included studies had obtained regulatory approval from agencies such as the United States Food and Drug Administration (FDA; Silver Spring, Maryland USA) or other relevant authorities. This limitation should be acknowledged when considering the broader applicability of these models in clinical practice.

## References

[ref1] Alrawashdeh A , Alqahtani S , Alkhatib ZI , et al. Applications and Performance of Machine Learning Algorithms in Emergency Medical Services: A Scoping Review. *Prehospital and Disaster Medicine*. Published online 2024:1-11. doi: 10.1017/S1049023X24000414 PMC11810483 38757150

