# Supplementary material for: Applications and Performance of Machine Learning Algorithms in Emergency Medical Services: A Scoping Review
Source: Prehosp Disaster Med. 2024 May 17;39(5):368–78. doi: 10.1017/S1049023X24000414 (PMC11810483; doi:10.1017/S1049023X24000414)
Supplement: Alrawashdeh et al. supplementary material [file S1049023X24000414sup001.docx]

Applications and performance of machine learning algorithms in emergency medical services: a scoping review

**Supplementary document**

**Preferred Reporting Items for Systematic reviews and Meta-Analyses extension for Scoping Reviews (PRISMA-ScR) Checklist**

| **SECTION** | **ITEM** | **PRISMA-ScR CHECKLIST ITEM** | **REPORTED ON PAGE #** |
| --- | --- | --- | --- |
| **TITLE** | | | |
| Title | 1 | Identify the report as a scoping review. | 1 |
| **ABSTRACT** | | | |
| Structured summary | 2 | Provide a structured summary that includes (as applicable): background, objectives, eligibility criteria, sources of evidence, charting methods, results, and conclusions that relate to the review questions and objectives. | 3 |
| **INTRODUCTION** | | | |
| Rationale | 3 | Describe the rationale for the review in the context of what is already known. Explain why the review questions/objectives lend themselves to a scoping review approach. | 4 |
| Objectives | 4 | Provide an explicit statement of the questions and objectives being addressed with reference to their key elements (e.g., population or participants, concepts, and context) or other relevant key elements used to conceptualize the review questions and/or objectives. | 5 |
| **METHODS** | | | |
| Protocol and registration | 5 | Indicate whether a review protocol exists; state if and where it can be accessed (e.g., a Web address); and if available, provide registration information, including the registration number. | 5 |
| Eligibility criteria | 6 | Specify characteristics of the sources of evidence used as eligibility criteria (e.g., years considered, language, and publication status), and provide a rationale. | 5-6 |
| Information sources* | 7 | Describe all information sources in the search (e.g., databases with dates of coverage and contact with authors to identify additional sources), as well as the date the most recent search was executed. | 5 |
| Search | 8 | Present the full electronic search strategy for at least 1 database, including any limits used, such that it could be repeated. | 5 |
| Selection of sources of evidence† | 9 | State the process for selecting sources of evidence (i.e., screening and eligibility) included in the scoping review. | 6 |
| Data charting process‡ | 10 | Describe the methods of charting data from the included sources of evidence (e.g., calibrated forms or forms that have been tested by the team before their use, and whether data charting was done independently or in duplicate) and any processes for obtaining and confirming data from investigators. | 6-7 |
| Data items | 11 | List and define all variables for which data were sought and any assumptions and simplifications made. | 6-7 |
| Critical appraisal of individual sources of evidence§ | 12 | If done, provide a rationale for conducting a critical appraisal of included sources of evidence; describe the methods used and how this information was used in any data synthesis (if appropriate). | NA |
| Synthesis of results | 13 | Describe the methods of handling and summarizing the data that were charted. | 7-8 |
| **RESULTS** | | | |
| Selection of sources of evidence | 14 | Give numbers of sources of evidence screened, assessed for eligibility, and included in the review, with reasons for exclusions at each stage, ideally using a flow diagram. | 8 |
| Characteristics of sources of evidence | 15 | For each source of evidence, present characteristics for which data were charted and provide the citations. | Supplement file |
| Critical appraisal within sources of evidence | 16 | If done, present data on critical appraisal of included sources of evidence (see item 12). | NA |
| Results of individual sources of evidence | 17 | For each included source of evidence, present the relevant data that were charted that relate to the review questions and objectives. | 8-11 |
| Synthesis of results | 18 | Summarize and/or present the charting results as they relate to the review questions and objectives. | 10-12 |
| **DISCUSSION** | | | |
| Summary of evidence | 19 | Summarize the main results (including an overview of concepts, themes, and types of evidence available), link to the review questions and objectives, and consider the relevance to key groups. | 12 |
| Limitations | 20 | Discuss the limitations of the scoping review process. | 17 |
| Conclusions | 21 | Provide a general interpretation of the results with respect to the review questions and objectives, as well as potential implications and/or next steps. | 17-18 |
| **FUNDING** | | | |
| Funding | 22 | Describe sources of funding for the included sources of evidence, as well as sources of funding for the scoping review. Describe the role of the funders of the scoping review. | 18 |

JBI = Joanna Briggs Institute; PRISMA-ScR = Preferred Reporting Items for Systematic reviews and Meta-Analyses extension for Scoping Reviews.

* Where *sources of evidence* (see second footnote) are compiled from, such as bibliographic databases, social media platforms, and Web sites.

† A more inclusive/heterogeneous term used to account for the different types of evidence or data sources (e.g., quantitative and/or qualitative research, expert opinion, and policy documents) that may be eligible in a scoping review as opposed to only studies. This is not to be confused with *information sources* (see first footnote).

‡ The frameworks by Arksey and O’Malley (6) and Levac and colleagues (7) and the JBI guidance (4, 5) refer to the process of data extraction in a scoping review as data charting*.*

§ The process of systematically examining research evidence to assess its validity, results, and relevance before using it to inform a decision. This term is used for items 12 and 19 instead of "risk of bias" (which is more applicable to systematic reviews of interventions) to include and acknowledge the various sources of evidence that may be used in a scoping review (e.g., quantitative and/or qualitative research, expert opinion, and policy document).

*From:* Tricco AC, Lillie E, Zarin W, O'Brien KK, Colquhoun H, Levac D, et al. PRISMA Extension for Scoping Reviews (PRISMAScR): Checklist and Explanation. Ann Intern Med. 2018;169:467–473. [doi: 10.7326/M18-0850](http://annals.org/aim/fullarticle/2700389/prisma-extension-scoping-reviews-prisma-scr-checklist-explanation).

**Table S1. Results of the search strategy from the four electronic databases.**

| No | Search strategy items | **Medline complete** | **CINAHL** | **Computer and Applied Science** | **Scopus** |
| --- | --- | --- | --- | --- | --- |
| S1 | (MH "Machine Learning+") OR(MH "Artificial Intelligence+") OR (MH "Unsupervised Machine Learning")OR (MH "Supervised Machine Learning+") OR (MH "Deep Learning") OR (MH "Neural Networks, Computer+") OR (MH" Natural Language Processing" | 142,643 | 27,514 | 6,796 | -- |
| S2 | TX "Machine learning" OR "deep learning" OR "neural network?" OR "support vector machine" OR "random forest" OR "decision trees" OR "nearest neighbors" OR "k?means" OR "na#ve bayes" OR "hierarchal clustering" OR "anomaly detection" OR "component analysis" OR "apriori algorithm" OR "reinforcement learning" OR "q? learning" OR "adversarial learning" OR "policy gradient" OR "policy optimi?ation" OR "natural language processing" OR "supervised W2 learning" OR "un#supervised W2 learning" OR "artificial intelligen*" OR perceptron OR “Dimensionality Reduction” OR “ensemble learning” OR "discriminant analysis" | 351,852 | 51,274 | 261,284 | 2,123,955 |
| S3 | (MH "Emergency Medical Services+") OR (MH "Emergency Medical Dispatch") OR (MH "Emergency Medical Service Communication Systems") OR (MH "Emergency Medical Technicians")OR (MH "Ambulances") OR (MH "Ambulance Diversion") | 117,701 | 121,413 | 325 | -- |
| S4 | TX "emergency medical service?" OR "out?of?hospital" OR "out-of-hospital" OR prehospital OR paramedic? OR ambulance OR "emergency medical technicians" OR "field triage | 50,825 | 44,919 | 1,928 | 255,316 |
| S5 | S1 OR S2 | 338,229 | 61,741 | 261,284 | -- |
| S6 | S5 OR S6 | 146,091 | 137,920 | 1,930 | -- |
| S7 | S5 AND S5 (Total) | 2,379 | 879 | 162 | 3,241 |

**Supplemental Table S2. Included Clinical studies.**

| **Author year** | **Study objective** | **Medical condition** | **Task** | **Region** | **No. of MLA** | **No. of cases input features** | **Type of input features** | **Best**  **ML** | **Performance metrics**  **AUC, Acc, Se, Sp** |
| --- | --- | --- | --- | --- | --- | --- | --- | --- | --- |
| Davis 2005 ^1^{Davis, 2005 #1}{Davis, 2005 #1} | Neural network analysis was performed to identify patients predicted to benefit from prehospital intubation. | Trauma | Tx | USA | S | 13625/ NA | Clinical | NN | 0.93, NA, 0.85, 0.864 |
| Yang 2005 ^2^ | To predict defibrillation outcomes to ROSC or No-ROSC using pre-shock ECG time series. | OHCA | CO | Europe | S | 110/ NA | ECG | NN | NA, 0.75, 0.83, 0.67 |
| Chen 2008 ^3^ | Present a classifier for use as a decision assist tool to identify a hypovolemic state in trauma patients during helicopter transport to a hospital, when reliable acquisition of vital-sign data may be difficult. | Trauma | Dx | USA | M | 898/ 5 | Clinical | Ensemble | 0.76, NA, 0.9, 0.4 |
| Davis 2008 ^4^ | To use ANN, SVM, and decision trees to explore the role of air medicine in TBI | Trauma | CO | USA | M | 11961/ NA | Clinical | NN | 0.92, NA, NA, NA |
| Krizmaric 2009 ^5^ | Intelligent analysis in predicting outcome of out-of-hospital cardiac arrest | OHCA | CO | Europe | M | 477/ 11 | ECG | DT | NA, 0.934, NA, NA |
| Scheetz 2009 ^6^ | To use crash scene data available to emergency responders to classify adults with moderate and severe injuries. | Trauma | Dx | USA | S | 74626/ 13 | Accident data | DT | NA, NA, 0.937, 0.7753 |
| Forberg 2012 ^7^ | To examine the ability of an artificial neural network (ANN) to safely reduce the number of ecgs transmitted by identifying patients without STEMI and patients not needing acute PCI. | CVD | Dx | Europe | S | 560/ 8 | ECG, Clinical | NN | NA, NA, 0.97, 0.68 |
| Jiang 2012 ^8^ | To use ensembled neural networks (ENN) to model survival rate for the patients with out-of-hospital cardiac arrest | OHCA | CO | Asia-Pacific | S | 4095/ 11 | Circumstantial, EMS data, Clinical | NN | NA, 0.89, NA, NA |
| Ayala 2014 ^9^ | Introduces a new approach to rhythm analysis during CPR that combines two strategies: a state-of-the-art CPR artifact suppression filter and a shock advice algorithm (SAA) designed to optimally classify the filtered signal. | OHCA | Dx | Europe | S | 247/ NA | ECG | SVM | NA, NA, NA, NA |
| Yunoki 2014 ^10^ | Propose an intelligent triage support system using a Bayesian network with the aim of improving the accuracy of call triage, the core of an emergency care support system. | Other | Dx | Asia-Pacific | S | 61927/ 41 | Audio | Bayesian | NA, 0.9487, NA, NA |
| Liu 2014 ^11^ | This study examined the utility of standard vital signs, HRV, HRC, and ML for predicting the need for lsis in trauma patients by comparing the performance of multivariate logistic regression identification versus ML technologies. | Trauma | Tx | USA | M | 104/ NA | ECG | NN | 0.99, NA, NA, NA |
| Goto 2014 ^12^ | To develop a simple and generally applicable bedside tool for predicting outcomes in children after cardiac arrest. | OHCA | CO | Asia-Pacific | S | 5379/ 3 | Clinical | DT | 0.88, NA, 0.797, 0.952 |
| Scerbo 2014 ^13^ | To validate the Random Forest computer model (RFM) as means of better triaging trauma patients to level 1 trauma centers. | Trauma | CO | USA | S | 1653/ 83 | Clinical | RF | NA, NA, 0.89, 0.42 |
| He 2015 ^14^ | Investigate whether combination of multiple VF features, by differen machine learning strategies could improve the prediction capacity of defibrillation outcome using a large multicenter database of OHCA patients. | OHCA | CO | Europe | M | 3828/ 16 | ECG | NN | 0.875, NA, 0.809, 0.809 |
| Figuera 2016 ^15^ | Detection of Shockable Rhythms in Automated External Defibrillators using machine learning | OHCA | Dx | USA | M | 29816/ 30 | ECG | SVM | NA, NA, 0.966, 0.988 |
| Rad 2016 ^16^ | Develop a system for automatic rhythm interpretation by using signal processing and machine learning algorithms. | OHCA | Dx | Europe | M | 302/ 32 | ECG, Clinical | Gaussian Mixture Model | NA, 0.68, NA, NA |
| Shandilya 2016 ^17^ | E hypothesize that a more complete picture of the cardiovascular system can be gained through non-linear dynamics and integration of multiple physiologic measures from biomedical signals. | OHCA | CO | USA | M | 153/ 20 | ECG | NN | 0.837, 0.774, NA, NA |
| Chicote 2016 ^18^ | Predict defibrillation success in OHCA scenarios | OHCA | CO | Europe | S | 163/ 6 | ECG | SVM | NA, NA, 0.804, 0.769 |
| He 2016 ^19^ | To find out whether combining VF features with additional attributes that related to the previous shock could enhance the prediction performance for subsequent shocks | OHCA | CO | Asia-Pacific | S | 528/ 3 | ECG | NN | 0.904, NA, 0.765, 0.9 |
| Rad 2017 ^20^ | Develop ECG-based algorithms for the retrospective and automatic classification of resuscitation cardiac rhythms. | OHCA | Dx | Europe | M | 1631/ 14 | ECG | NN | NA, 0.785, NA, NA |
| Yasuda 2017 ^21^ | The aim is to achieve higher overall performance and clarify the method for deciding the determination thresholds while simultaneously achieving both fail-safe determination and overall accuracy. | Other | Dx | Asia-Pacific | M | 328111/ 86 | Clinical | RF | NA, 0.923, NA, NA |
| Ruiz 2018 ^22^ | Design and evaluate a simple algorithm able to discriminate pulsatile rhythms from pulseless electrical activity during automated external defibrillator analysis intervals | OHCA | Dx | Europe | S | 302/ 6 | ECG | DT | NA, NA, 0.983, 0.984 |
| Chen 2018 ^23^ | Develop an artificial neural network (ANN) algorithm to predict LVO using prehospital accessible data | CVD | Dx | Asia-Pacific | S | 777/ 18 | Clinical | NN | 0.833, NA, NA, NA |
| Kim 2018 ^24^ | Developed a casualty classification model based on machine learning approaches for triage in mass casualty incidents by using a simplified consciousness score and vital signs that can be remotely monitored through wearable devices, without relying on medical practitioners. | Trauma | Dx | USA | M | 460865/ 5 | Clinical | NN | 0.89, NA, NA, NA |
| Thorpe 2018 ^25^ | To compare the diagnostic efficacy of two such candidate metrics: Velocity Asymmetry Index (VAI) and Velocity Curvature Index (VCI). Additionally, we investigate a simple decision tree combining both metrics. | CVD | Dx | USA | S | 66/ 66 | TCD waveform | DT | 0.94, 0.91, 0.94, 0.88 |
| Spangler 2019 ^26^ | Generating risk scores based on hospital outcomes using routinely collected prehospital data. | Other | Tx | Europe | S | 68668/ NA | Operation, Clinical | Ensemble | NA, NA, NA, NA |
| Chan 2019 ^27^ | Demonstrate that a support vector machine (SVM) can classify agonal breathing instances in real-time within a bedroom environment. | OHCA | Dx | USA | S | 162/ NA | Clinical | SVM | 0.9993, NA, 0.9724, 0.9951 |
| Harford 2019 ^28^ | Develop a machine learning model to predict a patient’s Cerebral Performance Category (CPC) score given a set of intervention and intermediate outcomes during a cardiac arrest event. | OHCA | CO | USA | M | 2639/ 27 | Clinical | NN | NA, NA, 0.825, NA |
| Elola 2019 ^29^ | Develop a pulse detection algorithm based exclusively on the ECG acquired by defibrillation pads. | OHCA | Dx | USA | M | 3914/ NA | ECG | SVM | NA, NA, 0.976, 0.862 |
| Kwon 2019 ^30^ | Develop and validate a deep-learning-based out-of-hospital cardiac arrest prognostic system (DCAPS) for predicting neurologic recovery and survival to discharge. | OHCA | CO | Asia-Pacific | M | 36190/ 10 | Clinical | NN | 0.953, NA, 0.951, 0.797 |
| Blomberg 2019 ^31^ | Examining whether a machine learning framework could recognize out-of-hospital cardiac arrest from audio files of calls to the emergency medical dispatch center | OHCA | Dx | Europe | M | 918/ NA | Audio | Ensemble | NA, NA, 0.841, 0.973 |
| Picon 2019 ^32^ | Introduces a deep learning architecture based on 1D-CNN layers and a Long Short-Term Memory (LSTM) network for the detection of VF. | OHCA | Dx | Europe | S | NA/ 20 | ECG | NN | NA, 0.991, 0.997, 0.989 |
| Elola 2019 ^33^ | The detection of return of spontaneous circulation by evaluating the added value of capnography for the classification of PR/PEA during OHCA. | OHCA | Dx | USA | S | 426/ 9 | ECG, TI | RF | 0.92, NA, 0.966, 0.945 |
| Seki 2019 ^34^ | To establish a prognostication model for OHCA with presumed cardiac aetiology using an advanced machine learning technique | OHCA | CO | Asia-Pacific | S | 16452/ 58 | Clinical | RF | 0.958, NA, NA, NA |
| Coult 2019 ^35^ | To evaluated a comprehensive group of VF waveform measures with and without ongoing compressions to determine their performance under both conditions for predicting functionally-intact survival, the study’s primary outcome | OHCA | CO | USA | M | 1151/ 27 | ECG | SVM | 0.75, NA, NA, NA |
| Ivanović 2019 ^36^ | Validate whether combining VF features can enhance the prediction accuracy in comparison to single feature | OHCA | CO | Europe | M | 251/ 28 | ECG | RF | 0.828, 0.828, 0.903, 0.754 |
| Johnsson 2020 ^37^ | Detection of dependencies between Clinical variables in OHCA survivors and prediction of functional outcome | OHCA | CO | Europe | S | 932/ NA | Clinical | NN | 0.891, NA, NA, NA |
| Kang 2020 ^38^ | Develop and validate an artificial intelligence (AI) algorithm based on deep learning to predict the need for critical care during EMS. | Trauma | Tx | Asia-Pacific | S | 8981181/ NA | Clinical | NN | 0.867, NA, NA, NA |
| Tollinton 2020 ^39^ | Investigate whether machine learning approaches using features from such free text notes can improve prediction of unconscious patients who require conveyance. | Other | Dx | Europe | M | 87281/ 2 | Text | Ensemble | 0.64, NA, NA, NA |
| AlDury 2020 ^40^ | To investigate the relative importance of 16 well-recognized factors in OHCA at the time point of ambulance arrival, and before any interventions or medications were given | OHCA | CO | Europe | S | 45067/ 16 | Clinical | RF | NA, NA, NA, NA |
| Krasteva 2020 ^41^ | Optimize the hyperparameters of an end-to-end fully convolutional neural network architecture for shockable/nonshockable rhythm detection | OHCA | Dx | Europe | S | NA/ NA | ECG | NN | NA, NA, 0.996, 0.994 |
| Ivanović 2020 ^42^ | Predicting defibrillation success in out-of-hospital cardiac arrested patients using conventional macine learning approches and proposing a novel approach in which predictive features are automatically learned. | OHCA | CO | Europe | S | 251/ NA | ECG | NN | NA, 0.936, 0.988, 0.882 |
| Jaureguibeitia 2020 ^43^ | Proposing novel deep learning architectures for shock decision algorithms based on convolutional and residual networks. | OHCA | Dx | Europe | M | 4216/ NA | ECG | NN | NA, 0.986, 0.991, 0.985 |
| Polero 2020 ^44^ | The aim of this study was to demonstrate the ability of machine learning classifiers to diagnose and predict an ACS in patients who spontaneously consult the EMS with undifferentiated chest pain, during a 30-day follow- up period. | CVD | Dx | USA | S | 161/ 20 | Clinical | RF | 0.8991, 0.8441, 0.8552, 0.8588 |
| Alonso 2020 ^45^ | This study presents a new method for pulse detection during OHCA using the ECG and TI signals. | OHCA | Dx | USA | S | 1140/ 40 | ECG | SVM | NA, 0.926, 0.924, 0.93 |
| Duceau 2020 ^46^ | To build a prediction algorithm to assist prehospital triage of AAS. | CVD | Dx | Europe | M | 976/ NA | Clinical | Ensemble | 0.73, NA, 0.79, 0.79 |
| Elola 2020 ^47^ | To develop a machine learning model to predict rearrest. | OHCA | CO | USA | S | 162/ 21 | ECG | RF | 0.689, NA, 0.673, 0.673 |
| Prieto 2020 ^48^ | To develop and test a natural language processing method that would improve identification of potential OM from paramedic documentation. | Other | Dx | USA | S | 54359/ NA | Text | LR | 0.939, NA, NA, NA |
| Qiu 2020 ^49^ | To establish a computational algorithm to predict the injury severity, so as to improve the timeliness, appropriateness, and efficacy of medical care provided. | Trauma | Dx | Asia-Pacific | S | 37/ 84 | Accident | NN | 0.747, NA, NA, NA |
| Jaureguibeitia 2020 ^50^ | To determine whether an impedance-based algorithm can accurately detect ventilations during concurrent mechanical chest compressions. | OHCA | Tx | USA | S | 423/ 14 | Clinical | RF | NA, NA, 0.963, NA |
| Wang 2021 ^51^ | To develop an NER model on paramedic text reports for Clinical audit. | Other | Tx | Asia-Pacific | M | 44211/ 17 | Text | NN | NA, NA, 0.976, NA |
| Stemerman 2021 ^52^ | To develope automated classification models to identify eligible patients for prehospital Clinical trials using EMS Clinical notes and compared model performance to manual review. | CVD | Dx | USA | M | 1209/ NA | Text | RF | NA, 0.92, 0.93, 0.96 |
| Manca 2021 ^53^ | Aimed to improve the assessment of alcohol burden on ambulance services in Scotland present estimates on the burden of alcohol on ambulance callouts in Scotland | Other | Dx | Europe | S | 5416/ NA | Text | RF | NA, 0.987, 0.996, 0.941 |
| Elola 2021 ^54^ | Determine circulation states during OHCA using the signals available in defibrillators. | OHCA | CO | USA | S | 210/ 37 | ECG | RF | NA, NA, 0.86, NA |
| Morris 2021 ^55^ | Develop a novel prediction model for hospital-triage that utilizes criteria available to the EMS provider to predict NEI-6 and the need for a trauma team activation | Other | Tx | USA | M | 22069/ 19 | Clinical | Ensemble | 0.85, NA, NA, NA |
| Hirano 2021 ^56^ | Develop and validate a machine learning-based prediction model of outcome for OHCA with an initial shockable rhythm | OHCA | CO | Asia-Pacific | M | 30049/ 19 | Clinical | NN | 0.888, 0.863, 0.919, 0.65 |
| Uchida 2021 ^57^ | Develop prehospital stroke scale with ML | CVD | Dx | Asia-Pacific | M | 3178/ 19 | Clinical | Ensemble | NA, 0.65, 0.47, 0.89 |
| Seo 2021 ^58^ | Developing and validated a machine learning-based system to predict good outcome in OHCA patients before ROSC. | OHCA | CO | Asia-Pacific | M | 5739/ 22 | ECG | Ensemble | 0.926, NA, 0.857, 0.865 |
| Candefjord 2021 ^59^ | Evaluate if methods employing machine learning and variables that can be assessed on the scene of accident has potential to amend field triage | Trauma | Dx | USA | M | 21589/ 6 | Accident | LR | 0.86, NA, NA, NA |
| Oka 2021 ^60^ | Our objectives were to determine the following. (1) which meteorological variable (among various meteorological variables) is the most important causative factor of heatstroke ? (2)how accurately can the observations be predicted when only meteorological variables are considered? | CVD | Dx | Asia-Pacific | S | NA/ NA | Clinical | RF | NA, NA, NA, NA |
| H 2021 ^61^ | Proposes a novel methodology based on ma- chine learning (ML) techniques to predict both the victims’ mortality and their need for transportation to health facil- ities using data gathered from the start of the emergency call until the Departmental Fire and Rescue Service of the Doubs (SDIS25) is notified. | OHCA | CO | Europe | S | 177883/ 7 | Clinical, EMS | LR | 0.79, NA, NA, NA |
| Lee 2021 ^62^ | Proposes a spatio-temporal demand model that incorporates batch arrivals of EMS calls. | OHCA | CO | Asia-Pacific | M | 105215/ NA | Spatiotemporal | NA | NA, NA, NA, NA |
| Urteaga 2021 ^63^ | The aim of this study was to develop a machine learning model to differentiate PEA with unfavorable (unpea) and favorable (fapea) evolution to ROSC. | OHCA | Dx | USA | S | 1921/ 17 | ECG | RF | 0.857, NA, 0.801, 0.767 |
| Byrsell 2021 ^64^ | To (1) examine whether a machine learning framework (ML) can increase the proportion of calls recognizing OHCA within the first minute compared with dispatchers, (2) present the performance of ML with different false positive rate (FPR) settings, (3) examine call characteristics influencing OHCA recognition. | OHCA | Dx | Europe | S | 851/ NA | Audio | Ensemble | NA, NA, 0.86, NA |
| Frigerio 2021 ^65^ | To assess the prediction accuracy when combined, and to clarify if they are correlated in out of hospital cardiac arrest' victims. | OHCA | CO | Europe | S | 112/ 2 | Clinical | SVM | 0.77, NA, 0.805, 0.688 |
| Larsson 2021 ^66^ | To assess whether, compared with logistic regression, the advanced machine learner xgboost (extreme Gradient Boosting) is associated with reduced prehospital trauma mistriage | Trauma | Dx | Europe | M | 813567/ 4 | Clinical | Ensemble | 0.725, NA, NA, NA |
| Yu 2021 ^67^ | To assist decision making on ambulance attendance and conveyance to a hospital using machine learning | Other | Tx | Europe | M | 25500/ NA | Clinical | NN | NA, 0.801, NA, NA |
| Hayasaka 2021 ^68^ | To create an AI model to classify intubation difficulty using deep learning (CNN), which connects the face image of a surgical patient and the actual difficulty of intubation. | Other | Tx | Asia-Pacific | S | 1043/ NA | Image | NN | 0.864, 0.805, 0.818, 0.833 |
| Isasi 2021 ^69^ | To demonstrate the first reliable shock decision algorithm during LDB compressions. | OHCA | Tx | Europe | M | 5813/ 38 | ECG | SVM | NA, 0.96, 0.921, 0.968 |
| Sashidhar 2021 ^70^ | To design and evaluate an ECG-based algorithm that predicts pulse presence with or without CPR | OHCA | CO | USA | M | 383/ NA | ECG | LR | 0.84, NA, NA, NA |
| Anthony 2021 ^71^ | To determine whether ML is a feasible option in classifying emergency call transcriptions, based off of the caller’s description of the patient. | CVD | Dx | Other | M | 93/ 107 | Text | SVM | NA, 0.95, NA, NA |
| Bouzid 2021 ^72^ | To develop a data-driven approach for ECG feature selection to build a Clinically relevant algorithm for real-time detection of culprit lesion | CVD | Dx | USA | S | 2400/ 557 | ECG | RF | 0.85, NA, 0.717, 0.847 |
| Mayampurath 2021 ^73^ | To develop a model that utilizes natural language processing of EMS reports and machine learning to improve prehospital stroke identification. | CVD | Dx | USA | S | 965/ NA | text | SVM | 0.73, NA, NA, NA |
| Nemeth 2021 ^74^ | To develop a phone- /tablet-based decision support system for prehospital tactical combat casualty care that collects physiologic and other Clinical data and uses machine learning to detect and differentiate shock manifestation. | Trauma | Dx | USA | M | 23744/ 70 | Clinical | LR | 0.85, NA, 0.73, 0.8 |
| Ferri 2021 ^75^ | To develop a predictive model to aid non-Clinical dispatchers to classify emergency medical call incidents by their life-threatening level (yes/no), admissible response delay (undelayable, minutes, hours, days) and emergency system jurisdiction (emergency system/primary care) in real time. | Trauma | Dx | Europe | M | 1244624/ NA | Circumstantial, demo, Clinical, free text | NN | NA, 0.771, NA, NA |
| Chin 2021 ^76^ | To develop an AI model for detecting a caller’s emotional state during out-of-hospital cardiac arrest calls by processing Audio recordings of dispatch communications. | OHCA | Dx | Asia-Pacific | S | 337/ NA | Audio | SVM | NA, NA, 0.3876, 0.9829 |
| Coult 2021 ^77^ | To develop an algorithm to predict the short- and long-term outcomes of VF shock without requiring CPR interruption. The proposed algorithm could potentially provide the basis to inform patient-specific VF treatment decisions during resuscitation while supporting the best practice of high quality continuous CPR. | OHCA | CO | USA | S | 1151/ 13 | ECG | SVM | 0.75, NA, NA, NA |
| Tohira 2021 ^78^ | To develop machine learning models using case characteristics and features gained by natural language processing of electronic free-text data from the ambulance services ehrs to identify fall cases | Trauma | Dx | Asia-Pacific | M | 9447/ NA | Text | SVM | NA, NA, 0.84, NA |
| Hayashi 2021 ^79^ | To develope prehospital stroke prediction algorithms using a machine learning approach with high precision | CVD | Dx | Asia-Pacific | M | 1446/ 52 | Clinical | Ensemble | 0.98, 0.952, 0.986, 0.864 |
| Blomberg 2021 ^80^ | To examine how a machine learning model trained to identify OHCA and alert dispatchers during emergency calls affected OHCA recognition and response | OHCA | Dx | Europe | S | 169049/ NA | Audio | Ensemble | NA, NA, 0.85, 0.974 |
| Cheng 2021 ^81^ | To investigate whether a machine learning algorithm could detect complex dependencies between Clinical variables in emergency departments in OHCA survivors and perform reliable predictions of favorable neurologic outcomes | OHCA | CO | Asia-Pacific | M | 1071/ NA | Clinical | Ensemble | 0.956, NA, 0.875, 0.904 |
| Jekova 2021 ^82^ | To optimize the architecture of a computationally efficient end-to-end CNN models for shock advisory decision during CPR using real-life AED recordings in OHCA | OHCA | Dx | Europe | S | 2720/ NA | ECG | NN | 0.938, NA, 0.89, NA |
| Tamminen 2021 ^83^ | To show whether adding blood glucose to the National Early Warning Score (NEWS) parameters in a machine learning model predicts 30-day mortality more precisely than the standard NEWS in a prehospital setting | Other | Dx | Europe | S | 3632/ 8 | Clinical | RF | 0.758, NA, NA, NA |
| Li 2021 ^84^ | We aimed to develop a machine learning model for trauma mortality prediction using variables easy to obtain in the prehospital setting. | Trauma | CO | Asia-Pacific | M | 1816723/ NA | Clinical | NN | 0.921, 0.578, 0.951, 0.559 |
| Ajumobi 2022 ^85^ | Identification of non-fatal opioid overdose cases using 9-1-1 computer assisted dispatch and prehospital patient clinical record variables | Other | Dx | USA | S | NA/ NA | Clinical | RF | NA, NA, 0.759, 0.999 |
| Hasan 2022 ^86^ | The aim of this study was to develop and improve prediction models for identifying adverse health outcomes for patients with suspected COVID-19 in a pre-hospital setting. | Other | CO | Europe | M | NA/ 26 | Clinical | NN | 0.9, NA, NA, NA |
| Takeda 2022 ^87^ | The aim of this study was to investigate a predictive power for predicting ACS  using the machine learning-based prehospital algorithm. | CVD | Dx | Asia-Pacific | M | NA/ 17 | Clinical | Ensemble | 0.861, 0.803, 0.772, 0.821 |
| Lin 2022 ^88^ | The authors performed several tree-based algorithms and an association rules mining as data mining tools to find useful determinants for neurological outcomes in out-of-hospital cardiac arrest (OHCA) patients as well as to assess the effect of the first-aid  and basic characteristics in the EMS system | OHCA | CO | Asia-Pacific | M | NA/ NA | Clinical | RF | NA, 0.9119, NA, NA |
| Harris 2022 ^89^ | The purpose of this study was to use NLP to examine EMS clinician free-text narratives for characteristics associated with prehospital ROSC in pediatric OHCA. | OHCA | CO | USA | M | NA/ NA | Text | LR | 0.92, NA, NA, NA |
| Zhang 2022 ^90^ | To achieve the automatic classification of prehospital emergency records. This study considers a deep learning-based prehospital emergency record classification model (dl-per). | Other | Dx | Asia-Pacific | S | NA/ NA | Text | NN | NA, NA, NA, NA |
| Lammers 2022 ^91^ | To assess and compare multiple machine learning models for predicting patients at highest risk for massive transfusion on the battlefield | Trauma | Tx | USA | M | 22158/ 22 | Clinical, demo | RF | 0.984, 0.9598, NA, 0.949 |
| Thannhauser 2022 ^92^ | To assess the performance of a single-variable approach to distinguish ACO from non-ACO patients, using AMSA prior to the first defibrillation attempt. | OHCA | CO | Europe | S | 100/ 1 | ECG | SVM | 0.8, NA, NA, NA |
| Chin 2022^93^ | To build a machine learning–based model through text mining of emergency calls for the automated identification of severely injured patients after a road accident. | Trauma | Dx | Asia-Pacific | M | NA/ NA | Text | Bayesian | NA, 0.75, 0.68, 0.78 |
| Harford 2022 ^94^ | To determine whether incorporating community variables into a ML model of OHCA can increase predictive accuracy of survival with functional neurologic outcome | OHCA | CO | USA | S | NA/ NA | Clinical, community | NN | 0.908, NA, NA, NA |
| Zhang 2022 ^95^ | To develop a MLP model to predict SM in patients | CVD | Dx | Asia-Pacific | S | NA/ NA | Clinical | NN | 0.855, 0.9, 0.708, 0.918 |
| Abe 2022 ^96^ | To develop a prehospital triage system to stratify patients with head trauma according to trauma severity by using several machine learning techniques and to evaluate the predictive accuracy of these techniques. | Trauma | Dx | Asia-Pacific | M | NA/ 18 | Clinical | Ensemble | 0.8, NA, 0.74, 0.749 |
| Liu 2022 ^97^ | To develop an interpretable prehospital ROSC (P-ROSC) score for ROSC prediction based on patients with OHCA in Asia. | OHCA | CO | Asia-Pacific | M | NA/ 5 | Clinical | LR | 0.806, NA, NA, NA |
| Harford 2022 ^98^ | To develop ML models that efectively predict hospital’s practice to perform coronary angiography (CA) in adult patients after OHCA and subsequent neurologic outcomes. | OHCA | CO | USA | M | 2398/ 47 | Clinical | NN | 0.908, NA, NA, NA |
| Paulin 2022 ^99^ | To fnd out whether machine learning can be used in this context and to identify the predictors of subsequent events based on narrative texts of electronic patient care records (epcr). | Other | CO | Europe | S | NA/ NA | Text | NN | 0.654, NA, NA, NA |
| Chen 2022 ^100^ | To implement an all-day online artificial intelligence (AI)-assisted detection of ST-elevation myocardial infarction (STEMI) by prehospital 12-lead electrocardiograms (ecgs) to facilitate patient triage for timely reperfusion therapy. | CVD | Dx | Asia-Pacific | S | NA/ NA | ECG | NN | 0.997, 0.992, 0.9409, 0.994 |
| Kawai 2022 ^101^ | To improve OHCA success rates, this study assessed the prognostic interactions resulting from simultaneously modifying two prehospital factors using a trained machine learning model. | OHCA | CO | Asia-Pacific | M | NA/ 24 | Clinical | NN | 0.9399, NA, NA, NA |
| Park 2022 ^102^ | To train and validate the time to on-scene return of spontaneous circulation prediction models using time-to-event analysis among out-of-hospital cardiac arrest patients. | OHCA | CO | Asia-Pacific | M | 105215/ NA | Community, EMS | NN | 0.873, NA, NA, NA |
| HajebM 2022 ^103^ | We demonstrate a novel application of a deep convolutional neural network encoder-decoder (CNNED) method to suppress CPR artifact in near real-time, using only ECG data. | OHCA | Dx | USA | S | NA/ NA | ECG | NN | NA, NA, 0.909, 0.991399 |
| Choi 2022 ^104^ | We developed and tested predictive models for TBI that use machine learning algorithms using information that can be obtained in the prehospital stage. | Trauma | CO | Asia-Pacific | M | 1169/ NA | Clinical | NN | 0.799, NA, 0.803, 0.61 |
| Shi 2023 ^105^ | To develop a cardiac arrest prediction model using deep learning (CAPD) algorithm and to validate the developed algorithm by evaluating the change in out-of-hospital cardiac arrest patient prognosis according to the increase in scene time interval (STI). | OHCA | CO | Asia-Pacific | M | NA/ 10 | Clinical | NN | 0.828, NA, 0.716, 0.768 |
| Bouzid 2023 ^106^ | We sought to compare the diagnostic performance of out-of-hospital and ED ECG and evaluate the incremental gain of artificial intelligence-augmented ECG analysis. | CVD | Dx | USA | S | NA/ 179 | ECG | RF | 0.83, NA, 0.75, 0.95 |
| deKoning 2023 ^107^ | Develop an AI model which would be able to predict ACS before patients visit the ED. | CVD | Dx | Europe | M | 7458/ 5 | Clinical | KNN | NA, NA, 0.995, 0.11 |
| Yoshida 2023 ^108^ | To develop a scale that predicts the need for surgical intervention in stroke patients. | CVD | Dx | Asia-Pacific | M | 1143/ 23 | Clinical | Ensemble | 0.802, 0.765, 0.719, 0.774 |
| Krasteva 2023 ^109^ | To present a novel deep learning algorithm for a sliding shock advisorydecision during cardiopulmonary resuscitation (CPR) and its performance evaluation as a function of the cumulative hands-off time. | OHCA | Dx | Europe | S | 13570/ 50 | ECG | NN | 0.9938, NA, 0.99, 1 |
| Alser 2023 ^110^ | Develop and test an artificial intelligence algorithm to predict critical care resource utilization. | Trauma | CO | USA | M | 41804/ NA | Clinical | NN | 0.848, NA, NA, NA |
| Yu 2023 ^111^ | To develop and validate an interpretable field triage scoring system for predicting mortality in pre-hospital patients in Asia. | Other | CO | Asia-Pacific | M | 26294/ NA | Clinical | NA | 0.938, NA, 0.888, 0.905 |
| HellsÃ©n 2023 ^112^ | Examine the 1-year prognosis of patients discharged from hospital after an OHCA. | OHCA | Dx | Europe | S | 5098/ 886 | Clinical | Ensemble | 0.73, NA, NA, NA |
| Bakidou 2023 ^113^ | Evaluate if an Artificial Intelligence (AI) based Clinical decision support system can identify severely injured trauma patients in the prehospital setting. | Trauma | CO | Europe | M | 47357/ 21 | Clinical | SVM | 0.89, 0.871, NA, NA |
| Gong 2023 ^114^ | To design a deep learning model to restore artifact-corrupted ECG signals during cardiopulmonary resuscitation (CPR) and provide shock/no-shock advice without needing additional reference signals. | OHCA | Dx | Asia-Pacific | S | 6113/ NA | ECG | NN | NA, 0.975, 0.959, 0.991 |
| Strum 2023 ^115^ | Utilize ML algorithms to predict hospital admission for adult patients arriving at the ED through paramedic transport with an emergent acuity triage score. | Other | CO | USA | M | NA/ 10 | Clinical | LR | 0.78, NA, 0.78, 0.37 |
| Spina 2023 ^116^ | We assessed the performance of the Clinical algorithms currently used in our PSAP (ie, operator-based interview) to identify patients that will test positive on SARS-cov- 2 rtpcr. | Other | Dx | Europe | M | 684481/ NA | Clinical | RF | 0.85, 0.85, 0.914, 0.442 |
| Kawai 2023 ^117^ | Predictive model of hemostatic need using factors that can be collected during helicopter emergency medical service (HEMS) interventions | Trauma | CO | Asia-Pacific | M | 251/ NA | Clinical | Ensemble | 0.8, NA, NA, NA |
| Coult 2023 ^118^ | We conducted a cohort study of VF out-of-hospital cardiac arrest to develop an ECG-based algorithm to predict patients with refractory VF. | OHCA | CO | USA | M | 1376/ NA | Clinical | NA | 0.85, NA, 0.63, 0.91 |
| Xu 2023 ^119^ | To develop machine learning models that can be adapted into primary and secondary MI triage tools and to externally validate these models using an independent population of injured patients. | Other | Dx | Europe | M | 193261/ 10 | Clinical | DT | 0.782, NA, 0.73, 0.739 |
| ValienteFernÃ¡ndez 2023 ^120^ | Comparison of the predictive ability of various machine learning algorithms (MLA) versus traditional prediction scales (TPS) for massive hemorrhage (MH) in patients with severe traumatic injury (STI) | Trauma | CO | Europe | M | NA/ NA | Clinical | RF | 0.99, NA, 0.91, 1 |
| Wang 2023 ^121^ | To develop a machine learning-based model for EMS ambulance dispatch triage in Singapore. | Other | Dx | Asia-Pacific | M | 361506/ NA | Clinical | RF | NA, 0.61, NA, NA |
| Kitano 2023 ^122^ | Aimed to create a prediction model specific to prehospital trauma care and to achieve greater accuracy with techniques of machine learning. | Trauma | CO | USA | M | NA/ 10 | Clinical | RF | 0.95, 0.97, 0.92, 0.92 |
| Chang 2023 ^123^ | Predict ROSC at the scene using prehospital input variables with time-adaptive cohort. | OHCA | CO | Asia-Pacific | M | 157654/ NA | Clinical | NA | NA, NA, NA, NA |
| Kim 2023 ^124^ | To develop a prediction model for transferring patients to an inappropriate hospital for suspected cardiovascular emergency diseases at the pre-hospital stage, using variables obtained from an integrated nationwide dataset, and to assess the performance of this model | CVD | Tx | Asia-Pacific | S | 94256/ 98 | Clinical | NN | 0.813, 0.739, 0.739, 0.739 |
| Tateishi 2023 ^125^ | Aimed to identify the prehospital factors that would affect favorable neurological survival in patients with witnessed OHCA and an initial shockable rhythm using the decision tree model. | OHCA | CO | Asia-Pacific | S | 86495/ NA | Clinical | DT | 0.844, 0.855, 0.668, 0.908 |
| CVD denotes cardiovascular diseases; OHCA denotes out-of-hospital cardiac arrest; Dx denotes diagnosis; CO denotes CO; Tx denotes ‎Tx; USA denotes United States of America; No MLA denotes number of machine learning algorithm; M denotes multiple; S denotes single; NA ‎denotes not available; ECG denotes electrocardiograph; TCD denotes transcranial doppler; ML denotes machine learning; SVM; support vector machine; RF ‎denotes random forest; NN denotes neural network; DT denotes decision tree; LR denotes logistic\linear regression; HMM denotes hidden Markov model; ‎AUC denotes area under the receiving operator curve; Acc denotes accuracy; Se denotes sensitivity; Sp denotes specificity.‎ | | | | | | | | | |

**Supplemental Table 3. The included operational studies.**

| **Author year** | **Study objective** | **Task** | **Region** | **No. of MLA** | **No. of cases/ input features** | **Type of input features** | **Best ML** | **Performance metrics** |
| --- | --- | --- | --- | --- | --- | --- | --- | --- |
| Setzler 2009 ^126^ | The objective of this study was to better forecast EMS call volumes, specifically, at a finer spatial and temporal granularity using ANN. | Ambulance allocation | USA | S | 181730/ 4 | Circumstantial, EMS data | NN | NA, NA, NA, NA |
| Grekousis 2014 ^127^ | Combine geographic information systems and neural networks for performing health emergency assessments and generating hazard maps that show areas that are potentially at high risk for emergencies | Ambulance allocation | Europe | S | 884/ NA | Spatial featues | NN | NA, NA, NA, NA |
| Chen 2015 ^128^ | Assess the service area of EMS after a disaster | Ambulance allocation | Asia-Pacific | S | NA/ NA | EMS data, transportation data | NN | NA, NA, NA, NA |
| Chen 2016 ^129^ | to investigate whether implementation of a coordinated digital-assisted program (CDAP) for Chinese hospitals can reduce the door-to-balloon (D2B) time for percutaneous coronary intervention (PCI) in acute chest pain patients in China | Ambulance allocation | Asia-Pacific | M | NA/ NA | EMS, temporal, GPS, metrological | NA | NA, NA, NA, NA |
| Bharsakade 2018 ^130^ | Locate an optimal number of EMS base locations so that the ambulance can achieve a response time of 8 minutes to a victim’s call for help | Ambulance allocation | Asia-Pacific | S | NA/ NA | Unsupervised ML | unsupervised ML | NA, NA, NA, NA |
| Grekousis 2019 ^131^ | Introducing a novel, three-level, spatial-based approach that identifies the geographical location of expected emergency events | Ambulance allocation | Asia-Pacific | S | 2851/ NA | EMS, geo, spatial feature | NN | NA, NA, NA, NA |
| Antunes 2019 ^132^ | An active learning metamodeling methodology to address the problem of policy analysis within the context of computationally expensive simulation models tested using an Emergency Medical Service (EMS) simulator. | Ambulance allocation | Europe | S | NA/ 92 | EMS | Bayesian | NA, NA, NA, NA |
| Jovanovic 2019 ^133^ | Developing a system for monitoring patient transport conditions with the comfort level classification, which is affected by the patient parameters | QA | Europe | M | 77/ 3 | Transportation, Clinical | SVM | NA, 0.9, NA, NA |
| Yang 2019 ^134^ | Proposes a simulation-based optimization method for ambulance allocation. | Ambulance allocation | Asia-Pacific | S | NA/ NA | Spatiotemporal | Gaussian Mixture Model | NA, NA, NA, NA |
| Mapuwei 2020 ^135^ | Examined the applicability of artificial neural network models in modelling univariate time series ambulance demand for short-term forecasting horizons in Zimbabwe | Ambulance allocation | Other | S | 108/ 7 | Ambulance demand | NN | NA, NA, NA, NA |
| Tran 2020 ^136^ | Investigates how to detect emergency vehicles such as ambulances, fire engines, and police cars based on their siren sounds. | Ambulance identification | Asia-Pacific | S | 26675/ NA | Audio, image | NN | NA, 0.9824, NA, NA |
| Dolejš 2020 ^137^ | Present a new travel time prediction model that is suitable for simple implementation using means that are currently available to EMS planners and decision makers and utilises real GPS dispatch logs data for speed training using ensemble learning methods | Ambulance allocation | Europe | S | NA/ NA | Traffic data | RF | NA, NA, NA, NA |
| Lin 2020 ^138^ | Proposing an original and novel approach that leverages machine learning tools and extraction of features based on the multi-nature insights of ambulance demands | Ambulance allocation | Asia-Pacific | M | NA/ NA | Spatial, demo, EMS | Ensemble | NA, 0.245, NA, NA |
| Redfield 2020 ^139^ | To link emergency medical services (EMS) electronic patient care reports (epcrs) to emergency department (ED) records. | QA | USA | S | 14032/ 6 | Clinical, text | LR | 0.99, NA, 0.994, NA |
| Martin 2021 ^140^ | A forecasting methodology that utilizes machine learning methods is proposed for producing, daily, hourly, and spatiotemporal call volume estimations at a degree of granularity in space and time that is practical and actionable. | Ambulance allocation | USA | S | 633417/ NA | EMS, spatiotemporal | NN | NA, NA, NA, NA |
| Xiong 2021 ^141^ | Develop and demonstrate a methodological framework of integrating transportation-sector data with health-related data to support various decision-making scenarios in transportation safety, emergency responses, and trauma-care triage. | route optimization | USA | S | 55000/ NA | Clinical, EMS, transportation, traffic | DT | 0.898, 0.884, 0.995, NA |
| Rashed 2021 ^142^ | Investigation of the correlation between environmental factors such as ambient temperature, absolute humidity, and the daily number of emergency ambulance dispatchs | Ambulance allocation | Asia-Pacific | S | NA/ NA | EMS, Metrological | NN | NA, NA, NA, NA |
| Jin 2021 ^143^ | Propose a bipartite graph convolutional neural network model to predict the EMS demand between hospital-region pairs. | Ambulance allocation | Asia-Pacific | S | 624062/ NA | Demo, Clinical, socioeconomic | NN | NA, 0.877, NA, NA |
| Cerna 2021^144^ | This paper proposes a novel two stage methodology based on machine learning (ML) models to forecast the turnaround time of each ambulance in a given time and hospital. | Ambulance deployment | Europe | M | 78777/ NA | Transportation, EMS | Ensemble | NA, 0.9702, NA, NA |
| Walker 2021 ^145^ | To derive and internally and externally validate machine-learning models to predict emergency ambulance patient door–to–off-stretcher wait times that are applicable to a wide variety of emergency departments. | Ambulance deployment | Asia-Pacific | M | 421894/ 18 | Clinical | RF | NA, NA, NA, NA |
| Ramgopal 2021 ^146^ | To develop and internally validate a metalearner algorithm to predict the hourly rate of emergency medical services (EMS) dispatches in an urban setting | Ambulance allocation | USA | M | 7364275/ NA | EMS, Metrological | Ensemble | NA, NA, NA, NA |
| Chu 2021 ^147^ | To develop dispatch rules for a network of defibrillator-carrying drones. | Ambulance deployment | USA | M | 3573/ 8 | Clinical, Spatial, EMS | NN | NA, 0.879, 0.935, 0.771 |
| Kumar 2021 ^148^ | To identify best Accedent Detection and Classification (ADC) machine learning model | Ambulance deployment | Asia-Pacific | M | NA/ NA | Accident features | Bayesian | NA, NA, 0.95, NA |
| Torres 2021 ^149^ | To predict the difference in travel time between the ground truth travel time provided by a GPS and the approximation offered by two mapping systems, Google Maps (GM) and Open Source Routing Machine (OSRM). | route optimization | Europe | S | 42363/ 9 | Spatial | RF | NA, 0.7164, NA, NA |
| Watanabe 2021 ^150^ | We tried to make prediction models for ambulance transports using the deep learning (DL) framework, Prediction One (Sony Network Communications Inc., Tokyo, Japan), with the meteorological and calendarial variables. | Ambulance allocation | Asia-Pacific | S | 5948/ NA | Metrological, EMS | NN | 0.972, NA, 0.937, 0.935 |
| Choi 2022 ^151^ | To develop and validate machine learning models for data entry error detection in a national out-of-hospital cardiac arrest (OHCA) prehospital patient care report database. | QA | Asia-Pacific | M | NA/ 19 | Clinical | LR | 0.95, NA, 0.83, 0.92 |
| Aldegheishem 2022 ^152^ | Is to present a driving assistance system for ambulances based on low-cost sensors, which proposes a recommended route to reach a destination when two or more possible routes can be taken. | route optimization | Other | S | NA/ NA | Spatiotemporal | NN | NA, 0.97, NA, NA |
| Charef 2022 ^153^ | Propose a hybrid approach consisting on a local approach using machine learning techniques to predict the congestion of different sections  of a map from an origin to a destination, and a global approach to suggest the fastest path to ambulance drivers in real time as they move in openstreetmap. | route optimization | Other | M | NA/ NA | Traffic, spatiotemporal | KNN | NA, 0.86, NA, NA |
| Patel 2022 ^154^ | Propose a system that detects an ambulance accurately and helps set up a makeshift emergency lane on the routes to be taken by it | Ambulance identification | Asia-Pacific | S | 2239/ NA | Audio | NN | NA, 0.972, NA, NA |
| Darwassh 2022 ^155^ | Proposes an ambulance vehicle routing approach in smart cities aims to take transfer the patients conœdentially, accurately, and quickly | route optimization | Other | S | NA/ NA | Accident, geo | NA | NA, NA, NA, NA |
| Li 2022 ^156^ | The primary objective of this study is to provide an AOD prediction model based on the current system status, hour of the day, and day of the week. With this information, decision-makers can be proactive with efforts to mitigate AOD. | Ambulance allocation | Asia-Pacific | M | NA/ NA | Historical, EMS | DT | NA, 0.9155, 0.9155, 0.6679 |
| Ceklic 2022 ^157^ | To determine how well the text sent to paramedics en-route to the traffic crash scene by the emergency medical dispatcher (EMD), in combination with dispatch codes, can predict the need for a L&S ambulance response to traffic crashes. | Ambulance deployment | Asia-Pacific | M | NA/ 9224 | Text | Ensemble | NA, 0.98, 0.98, NA |
| Rathore 2022 ^158^ | To propose a new vehicle routing and scheduling model equipped with novel features to ensure minimal response time using existing resources. | Ambulance allocation | Asia-Pacific | M | 9766/ NA | EMS, Clinical, geo, transportation | RF | NA, NA, NA, NA |
| Shimada-Sammori 2023 ^159^ | We hypothesized that machine learning algorithms using meteorological and chronological information can be used to accurately predict high OHCA incidence and help clinicians identify “high-risk” days for OHCA incidence. | Ambulance allocation | Asia-Pacific | M | NA/ NA | Meteorological | Ensemble | 0.906, 0.835, 0.848, 0.833 |
| Manguri 2023 ^160^ | To improve the performance accuracy of vehicle classification using certain preprocessing algorithms on the input images and testing various optimization methods. | Ambulance identification | Other | S | 6222/ NA | Image | NN | NA, 0.9844, NA, NA |
| Algamdi 2023 ^161^ | We build a framework utilizing CV technology to support decision-makers during the Hajj season | Ambulance identification | Other | S | 1234/ NA | Image | NN | NA, NA, NA, NA |
| Abreu 2023 ^162^ | To support decisions related to planning when, where and how many EMS resources are required. | Ambulance allocation | Europe | S | NA/ NA | EMS | NN | NA, 0.877, NA, NA |
| Ke 2023 ^163^ | Developed an effective method to predict the number of daily heat-related ambulance calls. | Ambulance allocation | Asia-Pacific | S | NA/ NA | Meteorological | Ensemble | NA, NA, NA, NA |
| Nithya 2024 ^164^ | To improve accuracy and reduce errors in vehicle sound classification. | Ambulance identification | Asia-Pacific | S | NA/ NA | Audio | NN | 0.98, 0.9866, 0.981, 0.994 |
| USA denotes United States of America; No. denotes number; MLA denotes machine learning algorithm; M denotes multiple; S denotes single; NA denotes not available; EMS denotes emergency medical services; ML denotes machine learning; SVM; support vector machine; RF denotes random forest; NN denotes neural network; DT denotes decision tree; LR denotes logistic\linear regression; HMM denotes hidden Markov model; KNN denotes K-nearest neighbour; AUC denotes area under the receiving operator curve; Acc denotes accuracy; Se denotes sensitivity; Sp denotes specificity. | | | | | | | | |

#

# References:

1. Davis DP, Peay J, Sise MJ, et al. The impact of prehospital endotracheal intubation on outcome in moderate to severe traumatic brain injury. *The Journal of trauma* 2005; 58: 933-939. 2005/05/28. DOI: 10.1097/01.ta.0000162731.53812.58.

2. Yang Z, Yang Z, Lu W, et al. A probabilistic neural network as the predictive classifier of out-of-hospital defibrillation outcomes. *Resuscitation* 2005; 64: 31-36. 2005/01/05. DOI: 10.1016/j.resuscitation.2004.07.002.

3. Chen L, McKenna TM, Reisner AT, et al. Decision tool for the early diagnosis of trauma patient hypovolemia. *Journal of biomedical informatics* 2008; 41: 469-478. 2008/02/08. DOI: 10.1016/j.jbi.2007.12.002.

4. Davis DP, Peay J, Good B, et al. Air medical response to traumatic brain injury: a computer learning algorithm analysis. *The Journal of trauma* 2008; 64: 889-897. 2008/04/12. DOI: 10.1097/TA.0b013e318148569a.

5. Krizmaric M, Verlic M, Stiglic G, et al. Intelligent analysis in predicting outcome of out-of-hospital cardiac arrest. *Computer methods and programs in biomedicine* 2009; 95: S22-32. 2009/04/04. DOI: 10.1016/j.cmpb.2009.02.013.

6. Scheetz LJ, Zhang J and Kolassa J. Classification tree modeling to identify severe and moderate vehicular injuries in young and middle-aged adults. *Artificial intelligence in medicine* 2009; 45: 1-10. 2008/12/19. DOI: 10.1016/j.artmed.2008.11.002.

7. Forberg JL, Khoshnood A, Green M, et al. An artificial neural network to safely reduce the number of ambulance ECGs transmitted for physician assessment in a system with prehospital detection of ST elevation myocardial infarction. *Scandinavian journal of trauma, resuscitation and emergency medicine* 2012; 20: 8. 2012/02/03. DOI: 10.1186/1757-7241-20-8.

8. Jiang Y-J, Ma MH-M, Sun W-Z, et al. Ensembled neural networks applied to modeling survival rate for the patients with out-of-hospital cardiac arrest. *Artificial Life and Robotics* 2012; 17: 241-244. DOI: 10.1007/s10015-012-0048-y.

9. Ayala U, Irusta U, Ruiz J, et al. A reliable method for rhythm analysis during cardiopulmonary resuscitation. *BioMed research international* 2014; 2014: 872470. 2014/06/05. DOI: 10.1155/2014/872470.

10. Yunoki S, Hamagami T, Oshige K, et al. High Accuracy of Call Triage Decision by Bayesian Network. *Electronics and Communications in Japan* 2014; 97: 62-69. DOI: <https://doi.org/10.1002/ecj.11439>.

11. Liu NT, Holcomb JB, Wade CE, et al. Utility of vital signs, heart rate variability and complexity, and machine learning for identifying the need for lifesaving interventions in trauma patients. *Shock (Augusta, Ga)* 2014; 42: 108-114. 2014/04/15. DOI: 10.1097/shk.0000000000000186.

12. Goto Y, Maeda T and Nakatsu-Goto Y. Decision tree model for predicting long-term outcomes in children with out-of-hospital cardiac arrest: a nationwide, population-based observational study. *Critical care (London, England)* 2014; 18: R133. 2014/06/29. DOI: 10.1186/cc13951.

13. Scerbo M, Radhakrishnan H, Cotton B, et al. Prehospital triage of trauma patients using the Random Forest computer algorithm. *The Journal of surgical research* 2014; 187: 371-376. 2014/02/04. DOI: 10.1016/j.jss.2013.06.037.

14. He M, Gong Y, Li Y, et al. Combining multiple ECG features does not improve prediction of defibrillation outcome compared to single features in a large population of out-of-hospital cardiac arrests. *Critical care (London, England)* 2015; 19: 425. 2015/12/15. DOI: 10.1186/s13054-015-1142-z.

15. Figuera C, Irusta U, Morgado E, et al. Machine Learning Techniques for the Detection of Shockable Rhythms in Automated External Defibrillators. *PloS one* 2016; 11: e0159654. 2016/07/22. DOI: 10.1371/journal.pone.0159654.

16. Rad AB, Engan K, Katsaggelos AK, et al. Automatic cardiac rhythm interpretation during resuscitation. *Resuscitation* 2016; 102: 44-50. 2016/02/20. DOI: 10.1016/j.resuscitation.2016.01.015.

17. Shandilya S, Kurz MC, Ward KR, et al. Integration of Attributes from Non-Linear Characterization of Cardiovascular Time-Series for Prediction of Defibrillation Outcomes. *PloS one* 2016; 11: e0141313. 2016/01/08. DOI: 10.1371/journal.pone.0141313.

18. Chicote B, Irusta U, Alcaraz R, et al. Application of Entropy-Based Features to Predict Defibrillation Outcome in Cardiac Arrest. *Entropy* *18(9)*(2016).

19. He M, Lu Y, Zhang L, et al. Combining Amplitude Spectrum Area with Previous Shock Information Using Neural Networks Improves Prediction Performance of Defibrillation Outcome for Subsequent Shocks in Out-Of-Hospital Cardiac Arrest Patients. *PloS one* 2016; 11: e0149115. 2016/02/11. DOI: 10.1371/journal.pone.0149115.

20. Rad AB, Eftestol T, Engan K, et al. ECG-Based Classification of Resuscitation Cardiac Rhythms for Retrospective Data Analysis. *IEEE transactions on bio-medical engineering* 2017; 64: 2411-2418. 2017/04/04. DOI: 10.1109/tbme.2017.2688380.

21. Yasuda T, Yamada Y, Hamatsu F, et al. A call triage support system for emergency medical service using multiple random forests. *IEEJ Transactions on Electrical and Electronic Engineering* 2017; 12: S67-S73. DOI: <https://doi.org/10.1002/tee.22567>.

22. Ruiz JM, Ruiz de Gauna S, González-Otero DM, et al. Circulation assessment by automated external defibrillators during cardiopulmonary resuscitation. *Resuscitation* 2018; 128: 158-163. 2018/05/08. DOI: 10.1016/j.resuscitation.2018.04.036.

23. Chen Z, Zhang R, Xu F, et al. Novel Prehospital Prediction Model of Large Vessel Occlusion Using Artificial Neural Network. *Frontiers in aging neuroscience* 2018; 10: 181. 2018/07/13. DOI: 10.3389/fnagi.2018.00181.

24. Kim D, You S, So S, et al. A data-driven artificial intelligence model for remote triage in the prehospital environment. *PloS one* 2018; 13: e0206006. 2018/10/24. DOI: 10.1371/journal.pone.0206006.

25. Thorpe SG, Thibeault CM, Canac N, et al. Decision Criteria for Large Vessel Occlusion Using Transcranial Doppler Waveform Morphology. *Frontiers in neurology* 2018; 9: 847. 2018/11/06. DOI: 10.3389/fneur.2018.00847.

26. Spangler D, Hermansson T, Smekal D, et al. A validation of machine learning-based risk scores in the prehospital setting. *PloS one* 2019; 14: e0226518. 2019/12/14. DOI: 10.1371/journal.pone.0226518.

27. Chan J, Rea T, Gollakota S, et al. Contactless cardiac arrest detection using smart devices. *npj Digital Medicine* 2019; 2: 52. DOI: 10.1038/s41746-019-0128-7.

28. Harford S, Darabi H, Del Rios M, et al. A machine learning based model for Out of Hospital cardiac arrest outcome classification and sensitivity analysis. *Resuscitation* 2019; 138: 134-140. 2019/03/20. DOI: 10.1016/j.resuscitation.2019.03.012.

29. Elola A, Aramendi E, Irusta U, et al. Deep Neural Networks for ECG-Based Pulse Detection during Out-of-Hospital Cardiac Arrest. *Entropy (Basel, Switzerland)* 2019; 21 2019/03/21. DOI: 10.3390/e21030305.

30. Kwon JM, Jeon KH, Kim HM, et al. Deep-learning-based out-of-hospital cardiac arrest prognostic system to predict COs. *Resuscitation* 2019; 139: 84-91. 2019/04/13. DOI: 10.1016/j.resuscitation.2019.04.007.

31. Blomberg SN, Folke F, Ersbøll AK, et al. Machine learning as a supportive tool to recognize cardiac arrest in emergency calls. *Resuscitation* 2019; 138: 322-329. 2019/01/22. DOI: 10.1016/j.resuscitation.2019.01.015.

32. Picon A, Irusta U, Álvarez-Gila A, et al. Mixed convolutional and long short-term memory network for the detection of lethal ventricular arrhythmia. *PloS one* 2019; 14: e0216756. 2019/05/21. DOI: 10.1371/journal.pone.0216756.

33. Elola A, Aramendi E, Irusta U, et al. Capnography: A support tool for the detection of return of spontaneous circulation in out-of-hospital cardiac arrest. *Resuscitation* 2019; 142: 153-161. DOI: <https://doi.org/10.1016/j.resuscitation.2019.03.048>.

34. Seki T, Tamura T and Suzuki M. Outcome prediction of out-of-hospital cardiac arrest with presumed cardiac aetiology using an advanced machine learning technique. *Resuscitation* 2019; 141: 128-135. 2019/06/21. DOI: 10.1016/j.resuscitation.2019.06.006.

35. Coult J, Blackwood J, Sherman L, et al. Ventricular Fibrillation Waveform Analysis During Chest Compressions to Predict Survival From Cardiac Arrest. *Circulation Arrhythmia and electrophysiology* 2019; 12: e006924. 2019/01/11. DOI: 10.1161/circep.118.006924.

36. Ivanović MD, Hannink J, Ring M, et al. Predicting defibrillation success in out-of-hospital cardiac arrested patients: Moving beyond feature design. *Artificial intelligence in medicine* 2020; 110: 101963. DOI: <https://doi.org/10.1016/j.artmed.2020.101963>.

37. Johnsson J, Björnsson O, Andersson P, et al. Artificial neural networks improve early outcome prediction and risk classification in out-of-hospital cardiac arrest patients admitted to intensive care. *Critical care (London, England)* 2020; 24: 474. 2020/08/01. DOI: 10.1186/s13054-020-03103-1.

38. Kang DY, Cho KJ, Kwon O, et al. Artificial intelligence algorithm to predict the need for critical care in prehospital emergency medical services. *Scandinavian journal of trauma, resuscitation and emergency medicine* 2020; 28: 17. 2020/03/07. DOI: 10.1186/s13049-020-0713-4.

39. Tollinton L, Metcalf AM and Velupillai S. Enhancing predictions of patient conveyance using emergency call handler free text notes for unconscious and fainting incidents reported to the London Ambulance Service. *International journal of medical informatics* 2020; 141: 104179. 2020/07/15. DOI: 10.1016/j.ijmedinf.2020.104179.

40. Al-Dury N, Ravn-Fischer A, Hollenberg J, et al. Identifying the relative importance of predictors of survival in out of hospital cardiac arrest: a machine learning study. *Scandinavian journal of trauma, resuscitation and emergency medicine* 2020; 28: 60. 2020/06/27. DOI: 10.1186/s13049-020-00742-9.

41. Krasteva V, Ménétré S, Didon JP, et al. Fully Convolutional Deep Neural Networks with Optimized Hyperparameters for Detection of Shockable and Non-Shockable Rhythms. *Sensors (Basel, Switzerland)* 2020; 20 2020/05/23. DOI: 10.3390/s20102875.

42. Ivanović MD, Hannink J, Ring M, et al. Predicting defibrillation success in out-of-hospital cardiac arrested patients: Moving beyond feature design. *Artificial intelligence in medicine* 2020; 110: 101963. 2020/12/01. DOI: 10.1016/j.artmed.2020.101963.

43. Jaureguibeitia X, Zubia G, Irusta U, et al. Shock Decision Algorithms for Automated External Defibrillators Based on Convolutional Networks. *IEEE Access* 2020; 8: 154746-154758. DOI: 10.1109/ACCESS.2020.3018704.

44. Polero LD, Garmendia CM, Echegoyen RE, et al. Predicción de riesgo de sufrir un síndrome coronario agudo mediante un algoritmo de Machine Learning (ANGINA). *Revista argentina de cardiología* 2020; 88: 9-13.

45. Alonso E, Irusta U, Aramendi E, et al. A machine learning framework for pulse detection during out-of-hospital cardiac arrest. *IEEE Access* 2020; 8: 161031-161041.

46. Duceau B, Alsac JM, Bellenfant F, et al. Prehospital triage of acute aortic syndrome using a machine learning algorithm. *Journal of British Surgery* 2020; 107: 995-1003.

47. Elola A, Aramendi E, Rueda E, et al. Towards the prediction of rearrest during out-of-hospital cardiac arrest. 2020; 22: 758.

48. Prieto JT, Scott K, McEwen D, et al. The detection of opioid misuse and heroin use from paramedic response documentation: machine learning for improved surveillance. *Journal of Medical Internet Research* 2020; 22: e15645.

49. Qiu J, Su S, Duan A, et al. Preliminary injury risk estimation for occupants involved in frontal crashes by combining computer simulations and real crashes. *Science progress* 2020; 103: 0036850420908750.

50. Jaureguibeitia X, Irusta U, Aramendi E, et al. Automatic detection of ventilations during mechanical cardiopulmonary resuscitation. *IEEE journal of biomedical and health informatics* 2020; 24: 2580-2588.

51. Wang H, Yeung WLK, Ng QX, et al. A weakly-supervised named entity recognition machine learning approach for emergency medical services Clinical audit. *International journal of environmental research and public health* 2021; 18: 7776.

52. Stemerman R, Bunning T, Grover J, et al. Identifying patient phenotype cohorts using prehospital electronic health record data. *Prehospital Emergency Care* 2022; 26: 78-88.

53. Manca F, Lewsey J, Waterson R, et al. Estimating the burden of alcohol on ambulance callouts through development and validation of an algorithm using electronic patient records. *International journal of environmental research and public health* 2021; 18: 6363.

54. Elola A, Aramendi E, Irusta U, et al. Multimodal algorithms for the classification of circulation states during out-of-hospital cardiac arrest. *IEEE Transactions on Biomedical Engineering* 2020; 68: 1913-1922.

55. Morris R, Karam BS, Zolfaghari EJ, et al. Need for Emergent Intervention within 6 Hours: A Novel Prediction Model for Hospital Trauma Triage. *Prehospital Emergency Care* 2022; 26: 556-565. DOI: 10.1080/10903127.2021.1958961.

56. Hirano Y, Kondo Y, Sueyoshi K, et al. Early outcome prediction for out-of-hospital cardiac arrest with initial shockable rhythm using machine learning models. *Resuscitation* 2021; 158: 49-56.

57. Uchida K, Kouno J, Yoshimura S, et al. Development of machine learning models to predict probabilities and types of stroke at prehospital stage: The Japan urgent stroke triage score using machine learning (JUST-ML). *Translational Stroke Research* 2022: 1-12.

58. Seo D-W, Yi H, Bae H-J, et al. Prediction of Neurologically Intact Survival in Cardiac Arrest Patients without Pre-Hospital Return of Spontaneous Circulation: Machine Learning Approach. *Journal of Clinical medicine* 2021; 10: 1089.

59. Candefjord S, Muhammad AS, Bangalore P, et al. On Scene Injury Severity Prediction (OSISP) machine learning algorithms for motor vehicle crash occupants in US. *Journal of Transport & Health* 2021; 22: 101124.

60. Oka K and Hijioka Y. Prediction of the number of heatstroke patients transported by ambulance in Japan’s 47 prefectures: proposal of heat acclimatization consideration. *Environmental Research Communications* 2021; 3: 125002.

61. Arcolezi HH, Cerna S, Couchot J-F, et al. Privacy-preserving prediction of victim’s mortality and their need for transportation to health facilities. *IEEE Transactions on Industrial Informatics* 2021; 18: 5592-5599.

62. Lee H and Lee T. Demand modelling for emergency medical service system with multiple casualties cases: K-inflated mixture regression model. *Flexible Services and Manufacturing Journal* 2021: 1-26.

63. Urteaga J, Aramendi E, Elola A, et al. A machine learning model for the prognosis of pulseless electrical activity during out-of-hospital cardiac arrest. *Entropy* 2021; 23: 847.

64. Byrsell F, Claesson A, Ringh M, et al. Machine learning can support dispatchers to better and faster recognize out-of-hospital cardiac arrest during emergency calls: A retrospective study. *Resuscitation* 2021; 162: 218-226. DOI: 10.1016/j.resuscitation.2021.02.041.

65. Frigerio L, Baldi E, Aramendi E, et al. End-tidal carbon dioxide (ETCO2) and ventricular fibrillation amplitude spectral area (AMSA) for shock outcome prediction in out-of-hospital cardiac arrest. Are they two sides of the same coin? *Resuscitation* 2021; 160: 142-149.

66. Larsson A, Berg J, Gellerfors M, et al. The advanced machine learner XGBoost did not reduce prehospital trauma mistriage compared with logistic regression: a simulation study. *BMC medical informatics and decision making* 2021; 21: 1-9.

67. Yu M, Kollias D, Wingate J, et al. Machine learning for predictive modelling of ambulance calls. *Electronics* 2021; 10: 482.

68. Hayasaka T, Kawano K, Kurihara K, et al. Creation of an artificial intelligence model for intubation difficulty classification by deep learning (convolutional neural network) using face images: an observational study. *Journal of Intensive Care* 2021; 9: 1-14.

69. Isasi I, Irusta U, Aramendi E, et al. Shock decision algorithm for use during load distributing band cardiopulmonary resuscitation. *Resuscitation* 2021; 165: 93-100.

70. Sashidhar D, Kwok H, Coult J, et al. Machine learning and feature engineering for predicting pulse presence during chest compressions. *Royal Society Open Science* 2021; 8: 210566.

71. Anthony T, Mishra AK, Stassen W, et al. The feasibility of using machine learning to classify calls to South African emergency dispatch centres according to prehospital diagnosis, by utilising caller descriptions of the incident. In: 2021 p.1107. MDPI.

72. Bouzid Z, Faramand Z, Gregg RE, et al. Novel ECG features and machine learning to optimize culprit lesion detection in patients with suspected acute coronary syndrome. *Journal of Electrocardiology* 2021; 69: 31-37. DOI: <https://doi.org/10.1016/j.jelectrocard.2021.07.012>.

73. Mayampurath A, Parnianpour Z, Richards CT, et al. Improving prehospital stroke diagnosis using natural language processing of paramedic reports. *Stroke* 2021; 52: 2676-2679.

74. Nemeth C, Amos-Binks A, Burris C, et al. Decision support for tactical combat casualty care using machine learning to detect shock. *Military medicine* 2021; 186: 273-280.

75. Ferri P, Sáez C, Félix-De Castro A, et al. Deep ensemble multitask classification of emergency medical call incidents combining multimodal data improves emergency medical dispatch. *Artificial intelligence in medicine* 2021; 117: 102088.

76. Chin K-C, Hsieh T-C, Chiang W-C, et al. Early recognition of a caller’s emotion in out-of-hospital cardiac arrest dispatching: An artificial intelligence approach. *Resuscitation* 2021; 167: 144-150.

77. Coult J, Rea TD, Blackwood J, et al. A method to predict ventricular fibrillation shock outcome during chest compressions. *Computers in Biology and Medicine* 2021; 129: 104136.

78. Tohira H, Finn J, Ball S, et al. Machine learning and natural language processing to identify falls in electronic patient care records from ambulance attendances. *Informatics for Health and Social Care* 2022; 47: 403-413.

79. Hayashi Y, Shimada T, Hattori N, et al. A prehospital diagnostic algorithm for strokes using machine learning: a prospective observational study. *Scientific Reports* 2021; 11: 20519. DOI: 10.1038/s41598-021-99828-2.

80. Blomberg SN, Christensen HC, Lippert F, et al. Effect of machine learning on dispatcher recognition of out-of-hospital cardiac arrest during calls to emergency medical services: a randomized Clinical trial. *JAMA network open* 2021; 4: e2032320-e2032320.

81. Cheng C-Y, Chiu IM, Zeng W-H, et al. Machine learning models for survival and neurological outcome prediction of out-of-hospital cardiac arrest patients. *BioMed research international* 2021; 2021.

82. Jekova I and Krasteva V. Optimization of end-to-end convolutional neural networks for analysis of out-of-hospital cardiac arrest rhythms during cardiopulmonary resuscitation. *Sensors* 2021; 21: 4105.

83. Tamminen J, Kallonen A, Hoppu S, et al. Machine learning model predicts short-term mortality among prehospital patients: A prospective development study from Finland. *Resuscitation Plus* 2021; 5: 100089.

84. Li Y, Wang L, Liu Y, et al. Development and validation of a simplified prehospital triage model using neural network to predict mortality in trauma patients: the ability to follow commands, age, pulse rate, systolic blood pressure and peripheral oxygen saturation (CAPSO) model. *Frontiers in Medicine* 2021; 8: 810195.

85. Ajumobi O, Verdugo SR, Labus B, et al. Identification of non-fatal opioid overdose cases using 9-1-1 computer assisted dispatch and prehospital patient Clinical record variables. *Prehospital emergency care* 2022; 26: 818-828.

86. Hasan M, Bath PA, Marincowitz C, et al. Pre-hospital prediction of adverse outcomes in patients with suspected COVID-19: Development, application and comparison of machine learning and deep learning methods. *Computers in Biology and Medicine* 2022; 151: 106024.

87. Takeda M, Oami T and Hayashi Y. Prehospital diagnostic algorithm for acute coronary syndrome using machine learning: a prospective observational study. Sci Rep. 2022; 12 (14593). *Research Square*.

88. Lin W-C, Huang C-H, Chien L-T, et al. Tree-Based Algorithms and Association Rule Mining for Predicting Patients’ Neurological Outcomes After First-Aid Tx for an Out-of-Hospital Cardiac Arrest During COVID-19 Pandemic: Application of Data Mining. *International Journal of General Medicine* 2022: 7395-7405.

89. Harris M, Crowe RP, Anders J, et al. Identification of factors associated with return of spontaneous circulation after pediatric out-of-hospital cardiac arrest using natural language processing. *Prehospital emergency care* 2023; 27: 687-694.

90. Zhang X, Zhang H, Sheng L, et al. DL-PER: Deep Learning Model for Chinese Prehospital Emergency Record Classification. *IEEE Access* 2022; 10: 64638-64649.

91. Lammers D, Marenco C, Morte K, et al. Machine learning for military trauma: novel massive transfusion predictive models in combat zones. *Journal of Surgical Research* 2022; 270: 369-375.

92. Thannhauser J, Nas J, van der Sluijs K, et al. Pilot study on VF-waveform based algorithms for early detection of acute myocardial infarction during out-of-hospital cardiac arrest. *Resuscitation* 2022; 174: 62-67.

93. Chin K-C, Cheng Y-C, Sun J-T, et al. Machine Learning–Based Text Analysis to Predict Severely Injured Patients in Emergency Medical Dispatch: Model Development and Validation. *Journal of Medical Internet Research* 2022; 24: e30210.

94. Harford S, Darabi H, Heinert S, et al. Utilizing community level factors to improve prediction of out of hospital cardiac arrest outcome using machine learning. *Resuscitation* 2022; 178: 78-84.

95. Zhang Z, Zhou D, Zhang J, et al. Multilayer perceptron-based prediction of stroke mimics in prehospital triage. *Scientific Reports* 2022; 12: 17994.

96. Abe D, Inaji M, Hase T, et al. A Prehospital Triage System to Detect Traumatic Intracranial Hemorrhage Using Machine Learning Algorithms. *JAMA Network Open* 2022; 5: e2216393-e2216393. DOI: 10.1001/jamanetworkopen.2022.16393.

97. Liu N, Liu M, Chen X, et al. Development and validation of an interpretable prehospital return of spontaneous circulation (P-ROSC) score for patients with out-of-hospital cardiac arrest using machine learning: A retrospective study. *EClinicalMedicine* 2022; 48.

98. Harford S, Del Rios M, Heinert S, et al. A machine learning approach for modeling decisions in the out of hospital cardiac arrest care workflow. *BMC Medical Informatics and Decision Making* 2022; 22: 1-9.

99. Paulin J, Reunamo A, Kurola J, et al. Using machine learning to predict subsequent events after EMS non-conveyance decisions. *BMC Medical Informatics and Decision Making* 2022; 22: 1-12.

100. Chen K-W, Wang Y-C, Liu M-H, et al. Artificial intelligence-assisted remote detection of ST-elevation myocardial infarction using a mini-12-lead electrocardiogram device in prehospital ambulance care. *Frontiers in cardiovascular medicine* 2022; 9: 1001982. DOI: 10.3389/fcvm.2022.1001982.

101. Kawai Y, Okuda H, Kinoshita A, et al. Visual assessment of interactions among resuscitation activity factors in out-of-hospital cardiopulmonary arrest using a machine learning model. *PloS one* 2022; 17: e0273787.

102. Park JH, Choi J, Lee S, et al. Use of time-to-event analysis to develop on-scene return of spontaneous circulation prediction for out-of-hospital cardiac arrest patients. *Annals of Emergency Medicine* 2022; 79: 132-144.

103. Hajeb-M S, Cascella A, Valentine M, et al. Enhancing the accuracy of shock advisory algorithms in automated external defibrillators during ongoing cardiopulmonary resuscitation using a deep convolutional Encoder-Decoder filtering model. *Expert Systems with Applications* 2022; 203: 117499.

104. Choi Y, Park JH, Hong KJ, et al. Development and validation of a prehospital-stage prediction tool for traumatic brain injury: a multicentre retrospective cohort study in Korea. *BMJ open* 2022; 12: e055918.

105. Shin SJ, Bae HS, Moon HJ, et al. Evaluation of optimal scene time interval for out-of-hospital cardiac arrest using a deep neural network. *The American Journal of Emergency Medicine* 2023; 63: 29-37.

106. Bouzid Z, Faramand Z, Martin-Gill C, et al. Incorporation of Serial 12-Lead Electrocardiogram With Machine Learning to Augment the Out-of-Hospital Diagnosis of Non-ST Elevation Acute Coronary Syndrome. *Annals of emergency medicine* 2023; 81: 57-69. DOI: 10.1016/j.annemergmed.2022.08.005.

107. de Koning E, van der Haas Y, Saguna S, et al. AI Algorithm to Predict Acute Coronary Syndrome in Prehospital Cardiac Care: Retrospective Cohort Study. *JMIR cardio* 2023; 7: e51375.

108. Yoshida Y, Hayashi Y, Shimada T, et al. Prehospital stroke-scale machine-learning model predicts the need for surgical intervention. *Scientific Reports* 2023; 13: 9135.

109. Krasteva V, Didon J-P, Ménétré S, et al. Deep Learning Strategy for Sliding ECG Analysis during Cardiopulmonary Resuscitation: Influence of the Hands-Off Time on Accuracy. *Sensors* 2023; 23: 4500.

110. Alser O, Dorken-Gallastegi A, Proaño-Zamudio JA, et al. Using the Field Artificial Intelligence Triage (FAIT) tool to predict hospital critical care resource utilization in patients with truncal gunshot wounds. *The American Journal of Surgery* 2023.

111. Yu JY, Heo S, Xie F, et al. Development and Asian-wide validation of the Grade for Interpretable Field Triage (GIFT) for predicting mortality in pre-hospital patients using the Pan-Asian Trauma Outcomes Study (PATOS). *The Lancet Regional Health–Western Pacific* 2023; 34.

112. Hellsén G, Rawshani A, Skoglund K, et al. Predicting recurrent cardiac arrest in individuals surviving Out-of-Hospital cardiac arrest. *Resuscitation* 2023; 184: 109678.

113. Bakidou A, Caragounis E-C, Andersson Hagiwara M, et al. On Scene Injury Severity Prediction (OSISP) model for trauma developed using the Swedish Trauma Registry. *BMC medical informatics and decision making* 2023; 23: 206.

114. Gong Y, Wei L, Yan S, et al. Transfer learning based deep network for signal restoration and rhythm analysis during cardiopulmonary resuscitation using only the ECG waveform. *Information Sciences* 2023; 626: 754-772.

115. Strum RP, Mowbray FI, Zargoush M, et al. Prehospital prediction of hospital admission for emergent acuity patients transported by paramedics: A population-based cohort study using machine learning. *PloS one* 2023; 18: e0289429.

116. Spina S, Gianquintieri L, Marrazzo F, et al. Detection of patients with COVID-19 by the emergency medical services in Lombardy through an operator-based interview and machine learning models. *Emergency Medicine Journal* 2023; 40: 810-820.

117. Kawai Y, Yamamoto K, Miyazaki K, et al. Explainable Prediction Model of the Need for Emergency Hemostasis Using Field Information During Physician-Staffed Helicopter Emergency Medical Service Interventions: A Single-Center, Retrospective, Observational Pilot Study. *Air Medical Journal* 2023.

118. Coult J, Yang BY, Kwok H, et al. Prediction of Shock-Refractory Ventricular Fibrillation During Resuscitation of Out-of-Hospital Cardiac Arrest. *Circulation* 2023.

119. Xu Y, Malik N, Chernbumroong S, et al. Triage in major incidents: development and external validation of novel machine learning-derived primary and secondary triage tools. *Emergency Medicine Journal* 2023.

120. Fernández MV, Fuentes CG, Moya FdPD, et al. Could machine learning algorithms help us predict massive bleeding at prehospital level? *Medicina Intensiva (English Edition)* 2023; 47: 681-690.

121. Wang H, Ng QX, Arulanandam S, et al. Building a Machine Learning-based Ambulance Dispatch Triage Model for Emergency Medical Services. *Health Data Science* 2023; 3: 0008.

122. Kitano S, Ogawa K, Igarashi Y, et al. Development of a Machine Learning Model to Predict Cardiac Arrest during Transport of Trauma Patients. *Journal of Nippon Medical School* 2023; 90: 186-193.

123. Chang H, Kim JW, Jung W, et al. Machine learning pre-hospital real-time cardiac arrest outcome prediction (PReCAP) using time-adaptive cohort model based on the Pan-Asian Resuscitation Outcome Study. *Scientific Reports* 2023; 13: 20344.

124. Kim JH, Kim B, Kim MJ, et al. Prediction of inappropriate pre-hospital transfer of patients with suspected cardiovascular emergency diseases using machine learning: a retrospective observational study. *BMC Medical Informatics and Decision Making* 2023; 23: 1-9.

125. Tateishi K, Saito Y, Yasufuku Y, et al. Prehospital predicting factors using a decision tree model for patients with witnessed out-of-hospital cardiac arrest and an initial shockable rhythm. *Scientific Reports* 2023; 13: 16180.

126. Setzler H, Saydam C and Park S. EMS call volume predictions: A comparative study. *Computers & Operations Research* 2009; 36: 1843-1851.

127. Grekousis G and Photis YN. Analyzing high-risk emergency areas with GIS and neural networks: The case of Athens, Greece. *The Professional Geographer* 2014; 66: 124-137.

128. Chen AY, Yu T-Y, Lu T-Y, et al. Ambulance service area considering disaster-induced disturbance on the transportation infrastructure. *Journal of Testing and Evaluation* 2015; 43: 479-489.

129. Chen AY, Lu TY, Ma MHM, et al. Demand Forecast Using Data Analytics for the Preallocation of Ambulances. *IEEE Journal of Biomedical and Health Informatics* 2016; 20: 1178-1187. DOI: 10.1109/JBHI.2015.2443799.

130. Bharsakade RS, Kulkarni OS, Afle AS, et al. Analysis of and modeling for emergency medical services facility location for road accidents on highway. *Int J Mech Prod Eng Res Dev* 2018; 8: 595-604.

131. Grekousis G and Liu Y. Where will the next emergency event occur? Predicting ambulance demand in emergency medical services using artificial intelligence. *Computers, Environment and Urban Systems* 2019; 76: 110-122.

132. Antunes F, Amorim M, Pereira FC, et al. Active learning metamodeling for policy analysis: Application to an emergency medical service simulator. *Simulation Modelling Practice and Theory* 2019; 97: 101947.

133. Jovanovic Z, Milosevic M, Jankovic D, et al. Comfort level classification during patients transport. *Technology and Health Care* 2019; 27: 61-77.

134. Yang W, Su Q, Huang SH, et al. Simulation modeling and optimization for ambulance allocation considering spatiotemporal stochastic demand. *Journal of Management Science and Engineering* 2019; 4: 252-265.

135. Mapuwei TW, Bodhlyera O and Mwambi H. Univariate time series analysis of short-term forecasting horizons using artificial neural networks: the case of public ambulance emergency preparedness. *Journal of Applied Mathematics* 2020; 2020: 1-11.

136. Tran V-T and Tsai W-H. Acoustic-based emergency vehicle detection using convolutional neural networks. *IEEE Access* 2020; 8: 75702-75713.

137. Dolejš M, Purchard J and Javorčák A. Generating a spatial coverage plan for the emergency medical service on a regional scale: Empirical versus random forest modelling approach. *Journal of Transport Geography* 2020; 89: 102889.

138. Lin AX, Ho AFW, Cheong KH, et al. Leveraging machine learning techniques and engineering of multi-nature features for national daily regional ambulance demand prediction. *International journal of environmental research and public health* 2020; 17: 4179.

139. Redfield C, Tlimat A, Halpern Y, et al. Derivation and validation of a machine learning record linkage algorithm between emergency medical services and the emergency department. *Journal of the American Medical Informatics Association* 2020; 27: 147-153.

140. Martin RJ, Mousavi R and Saydam C. Predicting emergency medical service call demand: A modern spatiotemporal machine learning approach. *Operations Research for Health Care* 2021; 28: 100285.

141. Xiong C, Yang M, Kozar R, et al. Integrating transportation data with emergency medical service records to improve triage decision of high-risk trauma patients. *Journal of Transport & Health* 2021; 22: 101106.

142. Rashed EA, Kodera S, Shirakami H, et al. Knowledge discovery from emergency ambulance dispatch during COVID-19: A case study of Nagoya City, Japan. *Journal of biomedical informatics* 2021; 117: 103743.

143. Jin R, Xia T, Liu X, et al. Predicting emergency medical service demand with bipartite graph convolutional networks. *Ieee Access* 2021; 9: 9903-9915.

144. Cerna S, Arcolezi HH, Guyeux C, et al. Machine learning-based forecasting of firemen ambulances’ turnaround time in hospitals, considering the COVID-19 impact. *Applied soft computing* 2021; 109: 107561.

145. Walker KJ, Jiarpakdee J, Loupis A, et al. Predicting ambulance patient wait times: a multicenter derivation and validation study. *Annals of Emergency Medicine* 2021; 78: 113-122.

146. Ramgopal S, Westling T, Siripong N, et al. Use of a metalearner to predict emergency medical services demand in an urban setting. *Computer methods and programs in biomedicine* 2021; 207: 106201.

147. Chu J, Leung KHB, Snobelen P, et al. Machine learning-based dispatch of drone-delivered defibrillators for out-of-hospital cardiac arrest. *Resuscitation* 2021; 162: 120-127.

148. Kumar N, Acharya D and Lohani D. An IoT-based vehicle accident detection and classification system using sensor fusion. *IEEE Internet of Things Journal* 2020; 8: 869-880.

149. Torres N, Trujillo L, Maldonado Y, et al. Correction of the travel time estimation for ambulances of the red cross Tijuana using machine learning. *Computers in Biology and Medicine* 2021; 137: 104798.

150. Watanabe O, Narita N, Katsuki M, et al. Prediction model of deep learning for ambulance transports in Kesennuma city by meteorological data. *Open Access Emergency Medicine* 2021: 23-32.

151. Choi DH, Park JH, Choi YH, et al. Machine learning analysis to identify data entry errors in prehospital patient care reports: a case study of a national out-of-hospital cardiac arrest registry. *Prehospital Emergency Care* 2024; 28: 14-22.

152. Aldegheishem A, Alrajeh N, Parra L, et al. Driving Assistance System for Ambulances to Minimise the Vibrations in Patient Cabin. *Electronics* 2022; 11: 3965.

153. Charef A, Jarir Z and Quafafou M. Smart System for Emergency Traffic Recommendations: Urban Ambulance Mobility. *International Journal of Advanced Computer Science and Applications* 2022; 13.

154. Patel R, Mange S, Mulik S, et al. AI based emergency vehicle priority system. *CCF Transactions on Pervasive Computing and Interaction* 2022; 4: 285-297.

155. Darwassh Hanawy Hussein T, Frikha M, Ahmed S, et al. BA-CNN: Bat algorithm-based convolutional neural network algorithm for ambulance vehicle routing in smart cities. *Mobile Information Systems* 2022; 2022.

156. Li M, Vanberkel P and Zhong X. Predicting ambulance offload delay using a hybrid decision tree model. *Socio-Economic Planning Sciences* 2022; 80: 101146.

157. Ceklic E, Ball S, Finn J, et al. Ambulance dispatch prioritisation for traffic crashes using machine learning: A natural language approach. *International journal of medical informatics* 2022; 168: 104886.

158. Rathore N, Jain PK and Parida M. A sustainable model for emergency medical services in developing countries: a novel approach using partial outsourcing and machine learning. *Risk management and healthcare policy* 2022: 193-218.

159. Shimada-Sammori K, Shimada T, Miura RE, et al. Machine learning algorithms for predicting days of high incidence for out-of-hospital cardiac arrest. *Scientific Reports* 2023; 13: 9950.

160. Manguri KHK and Mohammed AA. Emergency vehicles classification for traffic signal system using optimized transfer DenseNet201 model. *Indonesian Journal of Electrical Engineering and Computer Science* 2023; 32: 1058-1069.

161. Algamdi AM and Alghamdi HM. Instant Counting & Vehicle Detection during Hajj Using Drones. *Journal of Image and Graphics* 2023; 11: 204-211.

162. Abreu P, Santos D and Barbosa-Povoa A. Data-driven forecasting for operational planning of emergency medical services. *Socio-Economic Planning Sciences* 2023; 86: 101492.

163. Ke D, Takahashi K, Takakura Jy, et al. Effects of heatwave features on machine-learning-based heat-related ambulance calls prediction models in Japan. *Science of the total environment* 2023; 873: 162283.

164. Nithya TM, Dhivya P, Sangeethaa SN, et al. TB-MFCC multifuse feature for emergency vehicle sound classification using multistacked CNN–Attention BiLSTM. *Biomedical Signal Processing and Control* 2024; 88: 105688.
